# Inhibition of HSP90 distinctively modulates the global phosphoproteome of *Leishmania mexicana* developmental stages

**DOI:** 10.1128/spectrum.02960-23

**Published:** 2023-10-31

**Authors:** Exequiel O. Porta, Liqian Gao, Paul W. Denny, Patrick G. Steel, Karunakaran Kalesh

**Affiliations:** 1 Department of Chemistry, Durham University, Durham, United Kingdom; 2 School of Pharmaceutical Sciences, Shenzhen Campus of Sun Yat-sen University, Shenzhen, China; 3 Department of Biosciences, Durham University, Durham, United Kingdom; 4 School of Health and Life Sciences,Teesside University, Middlesbrough, United Kingdom; 5 National Horizons Centre, Darlington, United Kingdom; Weill Cornell Medicine, New York, New York, USA

**Keywords:** phosphorylation, *Leishmania*, protein kinases, HSP90, TMT labeling, LC-MS/MS, RNA helicase

## Abstract

Heat shock protein 90 (HSP90) is an evolutionarily conserved chaperone protein that plays a central role in the folding and maturation of a large array of client proteins. In the unicellular parasite *Leishmania*, the etiological agent of the neglected tropical disease leishmaniasis, treatment with HSP90 inhibitors leads to differentiation from promastigote to amastigote stage, resembling the effects of established environmental triggers, low pH and heat shock. This indicates a crucial role for HSP90 in the life cycle control of *Leishmania*. However, the underlying molecular mechanisms remain unknown. Using a combination of treatment with the classical HSP90 inhibitor tanespimycin, phosphoproteome enrichment, and tandem mass tag (TMT) labeling-based quantitative proteomic mass spectrometry (MS), we systematically characterized the perturbing effect of HSP90 inhibition on the global phosphoproteome of *Leishmania mexicana* across its life cycle stages and showed that the HSP90 inhibition causes substantially distinct molecular effects in promastigote and amastigote forms.While phosphorylation of HSP90 and its co-chaperone HSP70 was decreased in amastigote, the opposite effect was observed in promastigotes. Our results showed that kinase activity and microtubule motor activity are highly represented in the negatively affected phosphoproteins of the promastigotes, whereas ribosomal proteins, protein folding, and proton channel activity are preferentially enriched in the perturbed amastigote phosphoproteome. Additionally, cross-comparison of our results with HSP90 inhibition-affected RNA-binding proteins showed that RNA helicase domains were distinctively enriched among the upregulated amastigote phosphoproteins. In addition to providing robust identification and quantification of 1,833 phosphorylated proteins across three life cycle stages of *L. mexicana*, this study reveals the dramatically different ways the HSP90 inhibition stress modulates the phosphoproteome of the pathogenic amastigote and provides in-depth insight into the scope of selective molecular targeting in the therapeutically relevant amastigote stage.

**IMPORTANCE:**

In the unicellular parasites *Leishmania* spp., the etiological agents of leishmaniasis, a complex infectious disease that affects 98 countries in 5 continents, chemical inhibition of HSP90 protein leads to differentiation from promastigote to amastigote stage. Recent studies indicate potential role for protein phosphorylation in the life cycle control of *Leishmania*. Also, recent studies suggest a fundamentally important role of RNA-binding proteins (RBPs) in regulating the downstream effects of the HSP90 inhibition in *Leishmania*. Phosphorylation-dephosphorylation dynamics of RBPs in higher eukaryotes serves as an important on/off switch to regulate RNA processing and decay in response to extracellular signals and cell cycle check points. In the current study, using a combination of highly sensitive TMT labeling-based quantitative proteomic MS and robust phosphoproteome enrichment, we show for the first time that HSP90 inhibition distinctively modulates global protein phosphorylation landscapes in the different life cycle stages of *Leishmania*, shedding light into a crucial role of the posttranslational modification in the differentiation of the parasite under HSP90 inhibition stress. We measured changes in phosphorylation of many RBPs and signaling proteins including protein kinases upon HSP90 inhibition in the therapeutically relevant amastigote stage. This work provides insights into the importance of HSP90-mediated protein cross-talks and regulation of phosphorylation in *Leishmania*, thus significantly expanding our knowledge of the posttranslational modification in *Leishmania* biology.

## INTRODUCTION

*Leishmania* spp. are unicellular eukaryotic parasites that cause a complex spectrum of diseases in humans and animals (1). The parasite has a digenetic life cycle alternating between sandfly vectors and mammalian hosts adapting to changing environments via life cycle-specific expression of genes. The regulation of gene expression in *Leishmania* spp. is mostly posttranscriptional and involves processes such as mRNA processing, mRNA decay, protein synthesis, and posttranslational modifications (PTMs) (2). Among the different PTMs profiled during *Leishmania* differentiation, protein phosphorylation has been found to occur on proteins that correlate well to parasite differentiation through its life cycle, such as ribosomal proteins, cytoskeletal proteins, heat shock proteins, RNA-binding proteins (RBPs), protein kinases (PKs), and protein phosphatases (PPs) (3, 4). As the majority of *Leishmania* phosphoproteins identified to date are differentially expressed in the different life cycle stages (3, 5 – 7), it is believed that a complex interplay of protein phosphorylation and dephosphorylation events, facilitated by stage-specific PKs and PPs, plays a vital role in the intricate processes governing *Leishmania* differentiation.

Heat shock protein 90 (HSP90) has been previously identified as a downstream client of phosphorylation-mediated signaling in *Leishmania* spp. (8, 9). Interestingly, treatment with HSP90 inhibitors leads to dose- and time-dependent differentiation of *Leishmania* promastigotes to amastigotes in axenic cultures, suggesting a central role for the HSP90 in the life cycle control of the organism (10 – 12). We have recently shown that inhibition of HSP90 using the classical inhibitor tanespimycin causes a repression of ribosomal protein synthesis in *L. mexicana* promastigotes (13) and widespread perturbation of RNA-protein interactions in both promastigote and amastigote life cycle stages of *L. mexicana* (14). In particular, the RNA interactions of a substantial portion of the *L. mexicana* protein kinome were perturbed by the HSP90 inhibition (14). Phosphorylation and dephosphorylation of RBPs by the coordinated action of PKs and PPs are important regulatory mechanisms to control RNA processing and decay in response to cell cycle checkpoints and extracellular signals (15). To further investigate the dynamics of this interplay between phosphorylation and HSP90 action, we now describe a large-scale study capturing the global phosphoproteome of *L. mexicana*, characterizing its perturbations under the influence of the HSP90 inhibition. In order to accurately capture the modulations in the phosphoproteome, we combined for the first time the HSP90 inhibitor treatment in log-phase promastigote (LPP), stationary-phase promastigote (SPP) and axenic amastigote (AXA) life cycle stages of *L. mexicana* with phosphoproteome enrichment, followed by tandem mass tag (TMT) labeling (16)-based quantitative proteomic mass spectrometry (MS). This study significantly expands the protein phosphorylation landscape of *Leishmania* spp., providing robust identification of several thousands of phosphorylation sites and the modulatory effect of the HSP90 inhibition across the three different life cycle stages of this protozoan parasite.

## RESULTS

### Global changes in protein phosphorylation of the three life cycle stages of *Leishmania mexicana*

**Fig 1:**
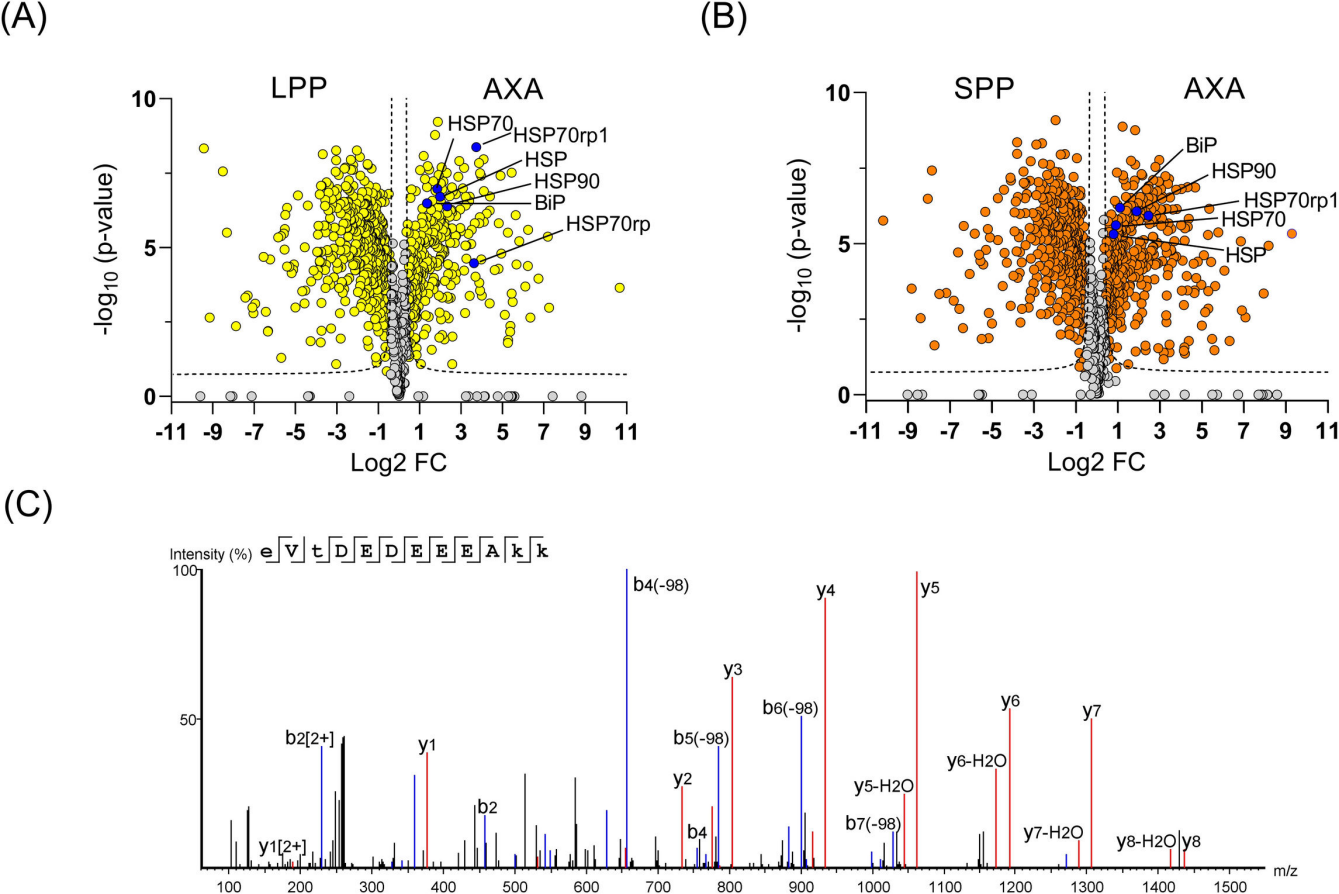
Global changes in the phosphoproteome of *L. mexicana* across its log-phase promastigote (LPP), stationary-phase promastigote (SPP), and axenic amastigote (AXA) life cycle stages profiled by phosphoproteome enrichment followed by tandem mass tag labeling-based quantitative proteomic mass spectrometry. All phosphoproteome enrichment experiments were performed in three biological replicates. (**A** and **B**) Volcano plots showing differential enrichment of phosphoproteins between LPP and AXA and between SPP and AXA, respectively. A modified *t-*test with permutation-based false discovery rate (FDR) statistics (250 permutations, FDR = 0.05) was applied to compare the quantitative differences in the phosphoproteins between the life cycle groups. Heat shock proteins (HSPs) that showed increased phosphorylation in the AXA are highlighted in blue filled circles. The following annotations were used for the different HSP family members: HSP70rp1: E9B125 (LmxM.29.2550); HSP70: E9B099 (LmxM.28.2770); HSP: E9ARS1 (LmxM.18.1370); HSP90: E9B3L2 (LmxM.32.0316, LmxM.32.0312, LmxM.32.0314); BiP: E9AZT9 (LmxM.28.1200); and HSP70rp: E9AYA3 (LmxM.26.1240), where the entries in brackets are the gene IDs. (**C**) MS/MS spectrum of Thr_216_ phosphorylated peptide from the *L. mexicana* HSP90.

TMT labeling-based quantitative proteomic MS of enriched phosphoproteome from LPP, SPP, and AXA life cycle stages of *L. mexicana* was performed in three biological replicates. A total of 1,833 phosphoproteins were identified with a minimum of two unique peptides across the three life cycle stages (Table S1). It should, however, be noted that, due to the inherent complexity in annotating the tandem mass (MS/MS) spectra of phosphopeptides, some of the low-scoring proteins may not be bona fide phosphoproteins and will require further validation studies to confirm their phosphorylation status. As shown in the volcano plots in Fig. 1A and B, we observed quantitative differences in the global phosphoproteome patterns across the life cycle stages (Tables S2 to S4). An increased phosphorylation of HSP90 and HSP70 in the amastigote stage of *Leishmania* compared to its promastigotes was previously reported (6). In agreement with these findings, our results show a quantitative increase in the phosphorylation of both proteins upon differentiation to AXA (Fig. 1A and B). A representative tandem mass spectrum of a phosphorylation site identification in the *L. mexicana* HSP90 is given in Fig. 1C. In addition to the previously reported phosphorylation at residues Thr_211_, Thr_216_, Ser_289_, Ser_371_, Ser_526_, Ser_594_, and Ser_595_ (8), our study identified five previously unknown phosphorylation sites at Ser_38_, Ser_48_, Thr_239_, Thr_256_, and Ser_477_ in the *L. mexicana* HSP90 (Fig. S1). Furthermore, this large-scale study revealed the phosphorylation sites and quantitative changes in the global phosphoproteome including important signaling kinases, RBPs, motor proteins, hydrolases, and ligases (Fig. S2) across the three life cycle stages of *L. mexicana* (Table S1 to S4).

### Functional analysis of the *L. mexicana* phosphoproteins

**Fig 2:**
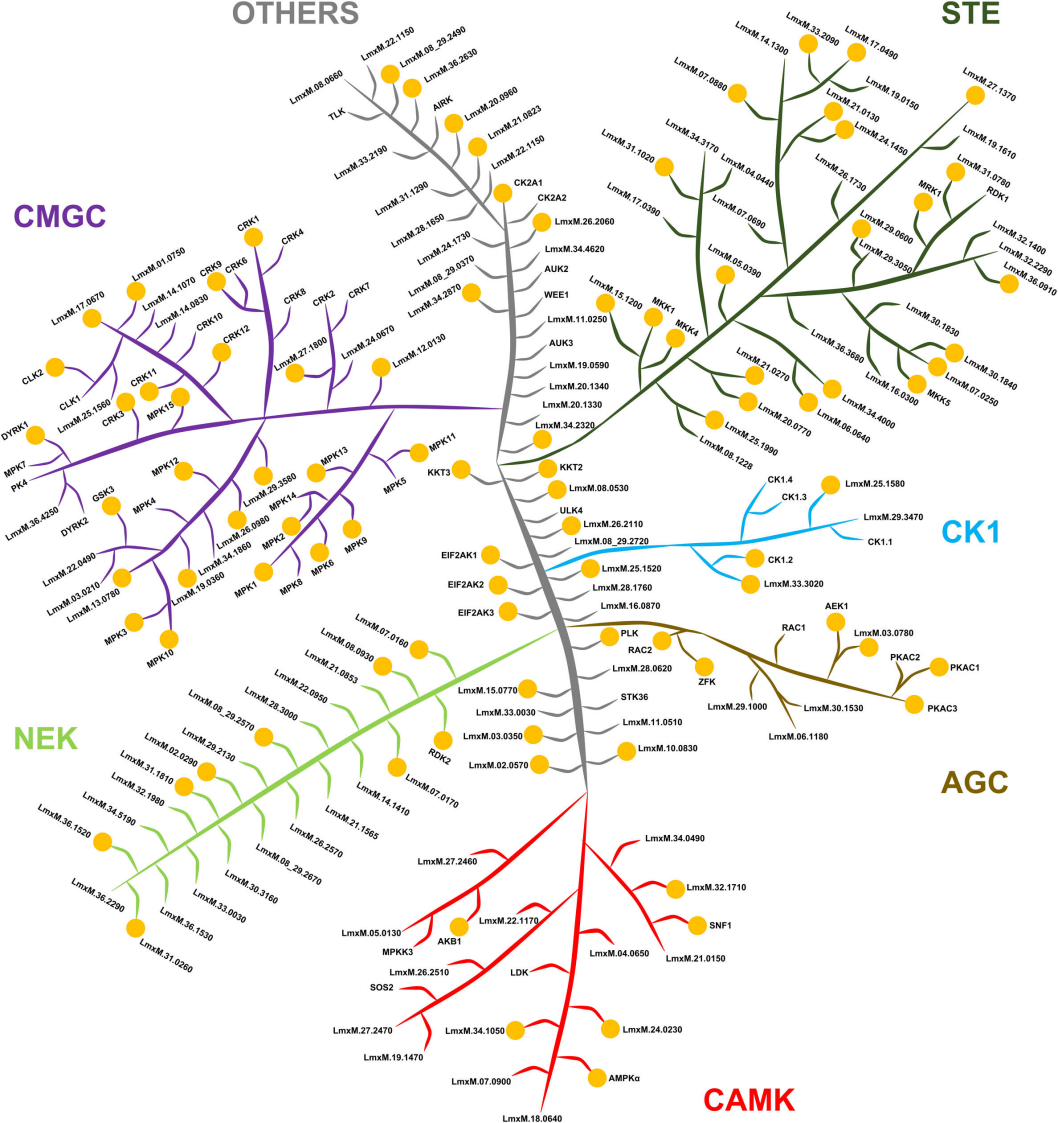
Phosphorylation in *L. mexicana* protein kinome. Classification of the protein kinases in *L. mexicana* according to their catalytic domain types, namely, GMGC, STE, NEK, CK1, AGC, CAMK, and others. The image has no phylogenetic significance and is for illustrative purpose only. The orange dots represent protein kinases in which phosphorylation was detected in this study.

Analysis of protein families and domains in the *L. mexicana* global phosphoproteome revealed protein kinase (PK) as the most enriched protein domain (Fig. S3). PK phosphorylation was found to be differentially regulated across the *L. mexicana* life cycle stages (Fig. S4). Phosphorylation of a set of PKs were found to be upregulated in the *L. mexicana* amastigote (Tables S2 to S4). These include MEKK-related kinase 1 (MRK1, LmxM.31.0120), LmxM.15.0770, LmxM.08_29.2570, casein kinase (LmxM.34.1010), LmxM.11.0060, rac serine/threonine kinase (LmxM.29.0800), MPK10 (LmxM.10.0200), MPK5 (LmxM.29.2910), 5′-AMP-activated PK (LmxM.08_29.2020), AGC essential kinase 1 (AEK1, LmxM.25.2340), PKAC3 (LmxM.18.1080), and MPK15 (LmxM.32.2070). Classification of *L. mexicana* PKs according to their catalytic domain conservation (17, 18) revealed that the majority of PKs in the CMGC and STE groups were phosphorylated (Fig. 2).

**Fig 3:**
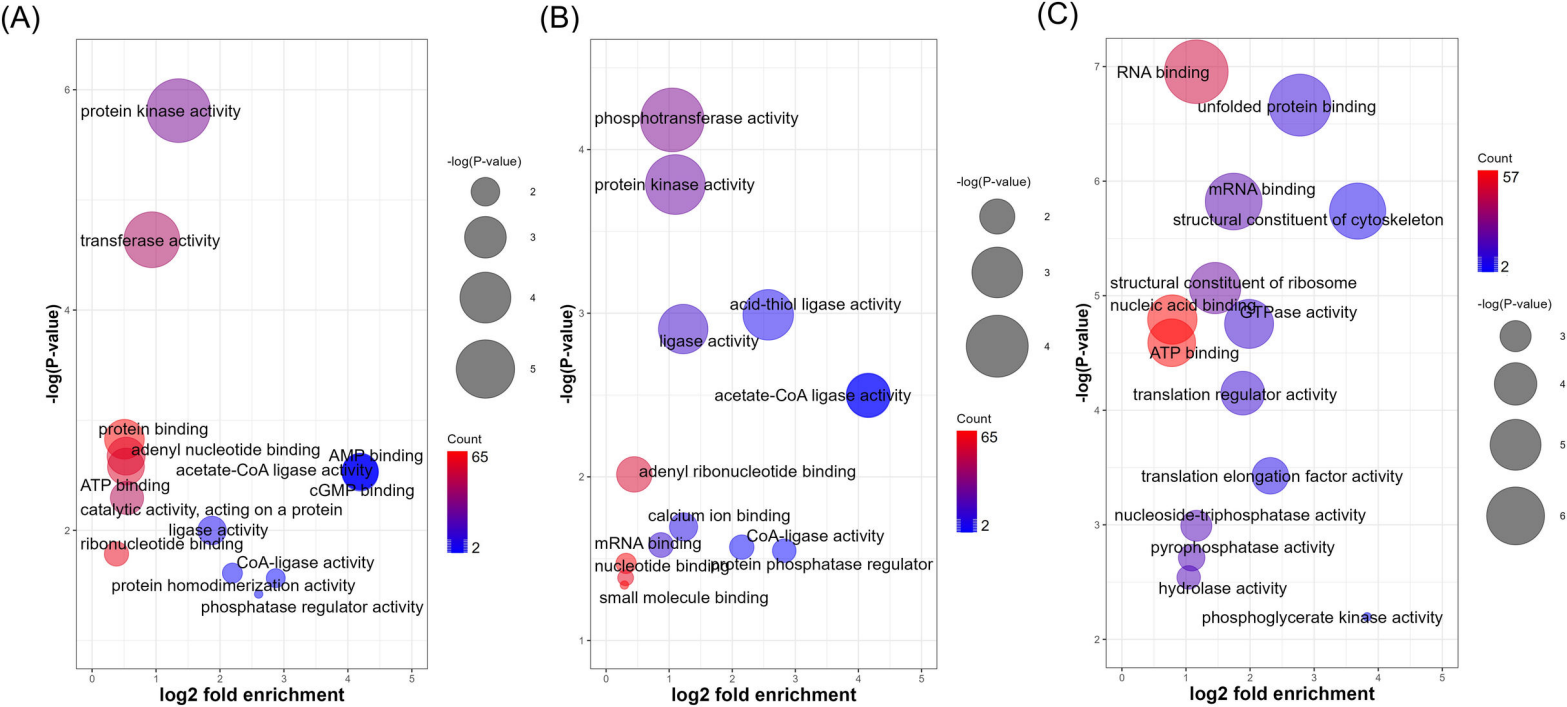
Molecular function gene ontology terms enriched in (**A**) LPP, (**B**) SPP, and (**C**) AXA phosphoproteins. CoA, coenzyme A.

Next, in order to gain more insight into the functional differences between the enriched phosphoproteins of the promastigotes and the amastigotes, we performed gene ontology (GO) analyses of the statistically significant differentially expressed phosphoproteins. The most enriched molecular function (MF) GO terms of the three life cycle stages are shown in Fig. 3. In contrast to the two promastigote stages, which showed a preferential enrichment of PK activity, the amastigote stage showed binding interactions, particularly RNA binding and unfolded protein binding, preferentially enriched among their phosphoproteins. Intriguing differences were also observed in the biological process (BP) GO terms of the phosphoproteins of the three life cycle stages (Fig. S5). In the LPP, the most enriched BP term was protein phosphorylation. However, in the SSP, along with protein phosphorylation, cytoplasmic translation was found to be a prominent BP. In a continuum, in the AXA, the translation became the most enriched BP term of the phosphoproteins. Similarly, striking differences were also observed in the cellular component (CC) GO terms of the phosphoproteins between the promastigotes and the amastigotes (Fig. S6). In both LPP and SSP, cilium and axoneme were the most enriched CC GO terms, suggesting potential involvement of protein phosphorylation in the structure and function of flagellum and plausible reliance of parasite cell motility on phosphorylation in the promastigotes. In contrast, enrichment of the supramolecular complex CC term in the AXA phosphoproteins suggests a life cycle-selective role for protein phosphorylation in the formation and function of large, multi-protein complexes involved in cellular processes such as protein translation.

### Physicochemical properties of the *L. mexicana* global phosphoproteome

**Fig 4:**
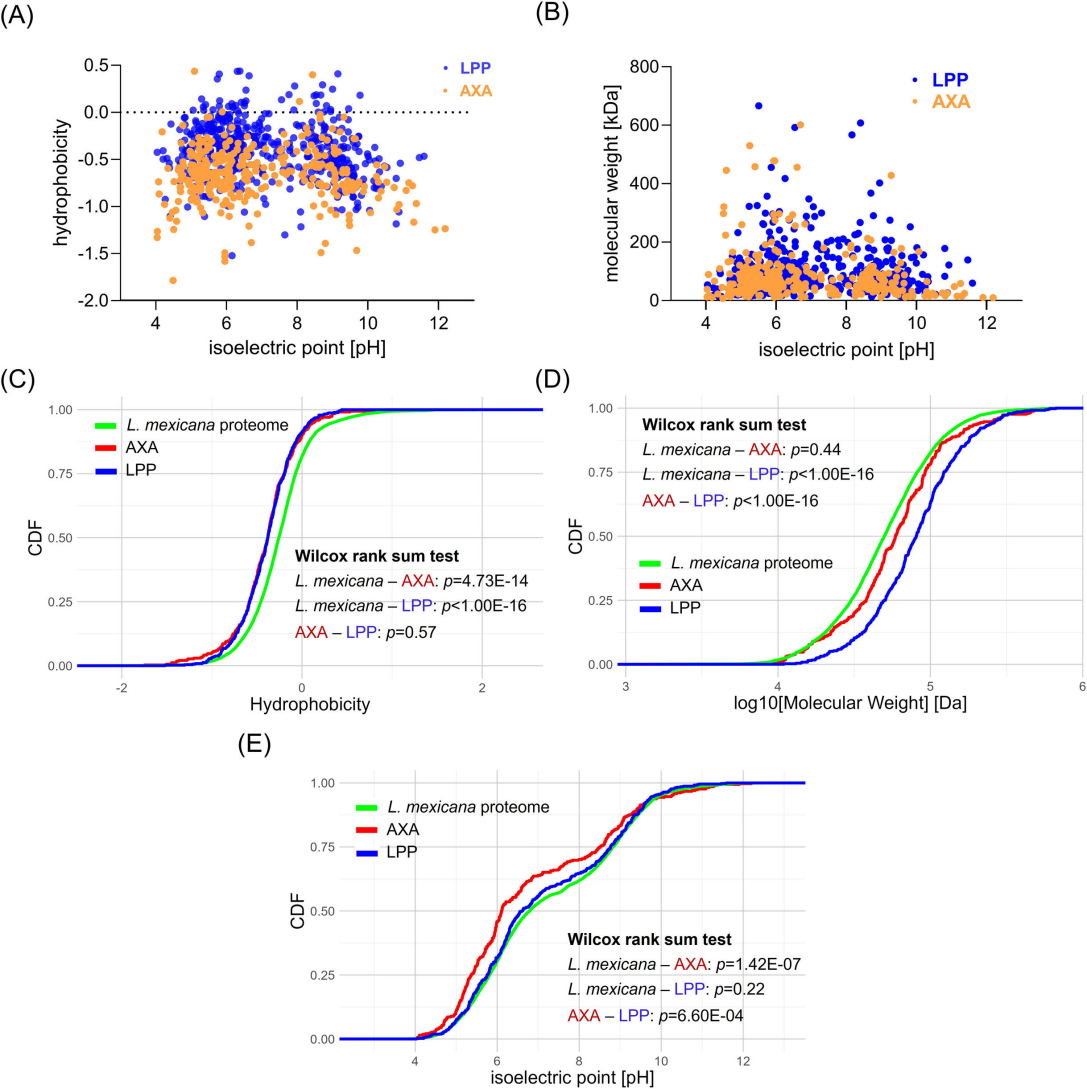
Physicochemical properties of *L. mexicana* phosphorylation substrates. (**A** and **B**) Scatter plots comparing hydrophobicity and isoelectric points and molecular weights and isoelectric points, respectively, of phosphorylation substrates in log-phase promastigote (LPP) and axenic amastigote (AXA) life cycle stages. (**C–E**) Cumulative distributions of hydrophobicity, molecular weights, and isoelectric points, respectively, in the AXA and LPP phosphorylation substrates and the entire *L. mexicana* proteome. Wilcox rank-sum test *P* values of the comparisons of *L. mexicana* total proteome vs. AXA phosphorylation substrates (*L. mexicana*-AXA) , *L. mexicana* total proteome vs. LPP phosphorylation substrates (*L. mexicana*-LPP) and AXA vs. LPP phosphorylation substrates (AXA–LPP) are shown. CDF, cumulative distribution function.

We then compared the physicochemical properties hydrophobicity, isoelectric point, and molecular weight of the *L. mexicana* phosphoproteome (Fig. 4A and B; Fig. S7). Cumulative distributions of the physicochemical properties in the LPP, SPP, and AXA phosphoproteins and the entire *L. mexicana* proteome revealed that the proteins undergoing phosphorylation in all life cycle stages are generally hydrophilic (Fig. 4C; Fig. S7C). The analysis also revealed that the LPP and SPP phosphorylation substrates are larger proteins (Fig. 4D; Fig. S7D), and the AXA phosphorylation substrates have comparatively more acidic isoelectric points (Fig. 4E; Fig. S7E).

### HSP90 inhibition differentially affects global protein phosphorylation of *L. mexicana* life cycle stages

**Fig 5:**
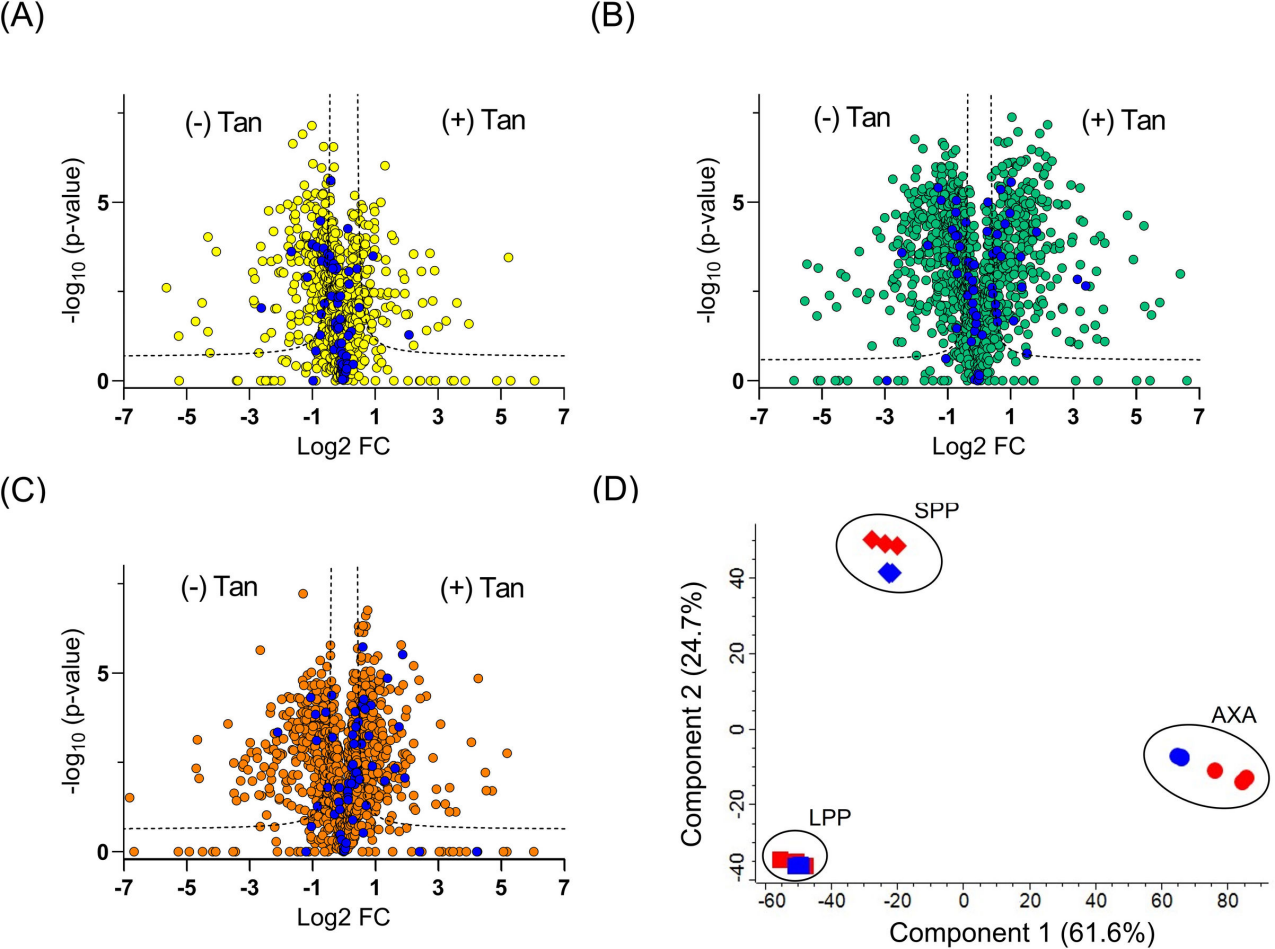
Effect of HSP90 inhibition via tanespimycin treatment on *L. Mexicana* phosphoproteome. (**A–C**) Volcano plots showing differential enrichment of phosphoproteins upon tanespimycin treatment (+) Tan and no treatment (−) Tan in log-phase promastigote (LPP), stationary-phase promastigote (SPP), and axenic amastigote (AXA) life cycle stages, respectively, profiled by phosphoproteome enrichment followed by TMT labeling-based quantitative proteomic MS. All experiments were performed in three biological replicates. A modified *t*-test with permutation-based FDR statistics (250 permutations, FDR = 0.05) was applied to compare the quantitative differences in the phosphoproteins between the tanespimycin-treated and non-treated groups. Protein kinases are highlighted in blue filled circles. (**D**) Principal component analysis of the phosphoproteins (blue) and the HSP90 inhibition affected phosphoproteins (red) in the three life cycle stages based on their relative quantification profiles.

Inhibition of HSP90 using tanespimycin caused substantial differences in the global phosphorylation in promastigotes and amastigotes (Fig. 5). While in the LPP, phosphorylation in the majority of PKs was found to decrease with HSP90 inhibition (Fig. 5A), the opposite trend was observed in the AXA (Fig. 5C). Interestingly, the overall changes in the protein kinome phosphorylation in the SPP were found to lie in between those of the LPP and the AXA (Fig. 5B). Principal component analysis of the phosphoproteins and the HSP90 inhibition-affected phosphoproteins based on their relative quantification profiles revealed clustering of the proteins according to the life cycle stages (Fig. 5D).

In the LPP, HSP90 inhibition negatively affected the phosphorylation of 623 proteins (Table S5). These phosphoproteins were enriched in the MFs (Fig. S8A), microtubule motor activity (*P* value, 5.68e−5) and kinase activity (*P* value, 9.76e−5). The top BPs (Fig. S8B) of these downregulated proteins were microtubule-based movement (*P* value, 1.53e−6) and chromatin assembly or disassembly (*P* value, 1.39e−5). The most enriched CCs (Fig. S8C) of these negatively affected phosphoproteins were cilium (*P* value, 4.77e−9) and cytoskeleton (*P* value, 3.63e−8). Tanespimycin treatment also led to statistically significant increased phosphorylation of a total of 236 phosphoproteins in the LPP (Table S5). The upregulated phosphoproteins in the LPPs were enriched in the MFs (Fig. S8A), anion binding (*P* value, 3.31e−6), and ribonucleotide binding (*P* value, 6.96e−6). The top BPs (Fig. S8B) of these upregulated phosphoproteins were microtubule-based process (*P* value, 9.54e−5) and protein folding (*P* value, 3.07e−4). Interestingly, tanespimycin treatment increased the phosphorylation of both HSP90 and HSP70 in the LPP (Fig. S9). Phosphorylation of Thr_216_ and Ser_526_ residues in the HSP90 was the most upregulated upon tanespimycin treatment (Fig. S10).The most enriched CCs (Fig. S8C) of the positively regulated phosphoproteins were cilium (*P* value, 7.73e−6) and eukaryotic translation initiation factor complex 4F (*P* value, 6.57e−5).

In the SPP, tanespimycin treatment negatively and positively affected the phosphorylation of 702 and 455 proteins respectively (Table S6). GO analyses of these HSP90 inhibition-affected phosphoproteins in the SPPs showed similar overall trends as that of the affected proteins in the LPP (Fig. S11). However, disparities in the cellular processes of the affected proteins were revealed between the promastigotes and the AXA. For example, in contrast to the promastigotes, tanespimycin treatment in the AXA was found to cause a decrease in the phosphorylation of both HSP90 and HSP70 (Fig. S9). In the AXA, the HSP90 inhibition negatively and positively affected the phosphorylation of 429 and 614 proteins, respectively (Table S7). The proteins that showed a decreased phosphorylation upon tanespimycin treatment in the AXA were highly enriched in the MFs (Fig. S11), structural constituent of ribosome (*P* value, 6.28e−16) and unfolded protein binding (*P* value, 1.44e−8). Among the most highly enriched BPs of these phosphoproteins were translation (*P* value, 8.45e^−16^) and protein folding (*P* value, 2.28e−10). The most enriched CC GO terms were cytosolic ribosome (*P* value, 5.14e−14) and cytosolic large ribosomal subunit (*P* value, 1.90e−12). The AXA proteins that showed an increased phosphorylation upon tanespimycin treatment were highly enriched in the MFs (Fig. S12), mRNA binding (*P* value, 7.38e-8), and RNA binding (*P* value, 4.56e−6). Among the most highly enriched BPs of these proteins were movement of cell or subcellular component (*P* value, 1.05e−3) and ribosomal large subunit assembly (*P* value, 2.08e−3). The most enriched CC GO terms of these upregulated AXA phosphoproteins were nucleolus (*P* value, 6.37e−9) and eukaryotic translation initiation factor complex 4F (*P* value, 5.12e−7).

### Correlation between the global phosphoproteome and protein-RNA interactions upon HSP90 inhibition in *L. mexicana*

We have recently demonstrated that HSP90 inhibition causes widespread perturbation of protein-RNA interactions in *L. mexicana* (14). As phosphorylation-dephosphorylation dynamics of RBPs in higher eukaryotes regulate RNA processing and decay in response to various signals (15), we set out to identify the RBPs among the tanespimycin-affected phosphoproteins in both amastigote and promastigote life cycle stages of *L. mexicana*. Because HSP90 inhibition predominantly downregulated protein-RNA interactions in both life cycle stages of *L. mexicana* (14), we compared the downregulated RBPs in our published data sets with both upregulated and downregulated phosphoproteins identified in this study.

**Fig 6:**
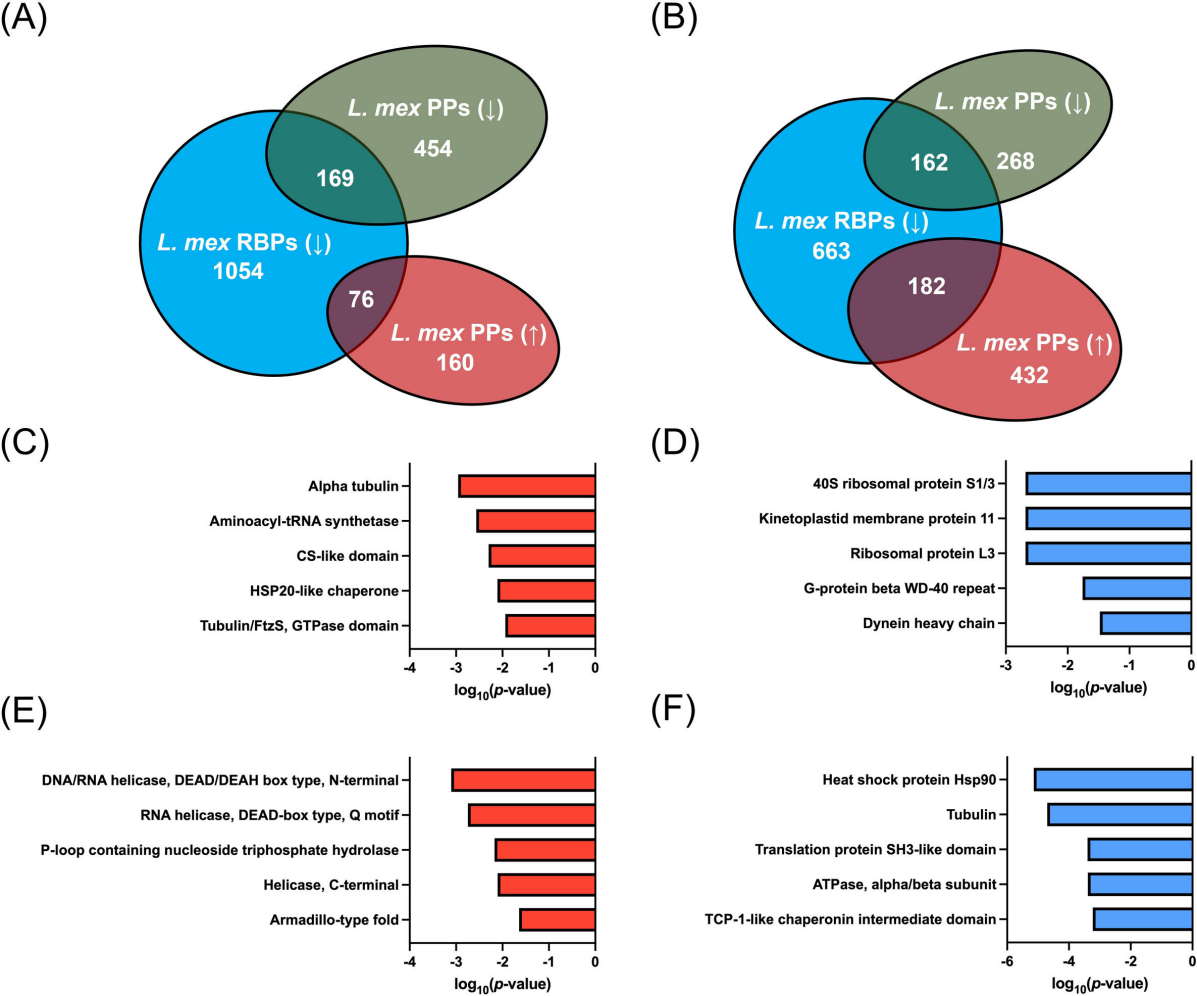
Correlation between RNA-binding proteins (RBPs) downregulated by HSP90 inhibition and the HSP90 inhibition-modulated phosphoproteins in *L. mexicana*. Venn diagrams showing comparison of the downregulated RBPs [*L. mex* RBPs (↓)] and upregulated [*L. mex* PPs (↑)] and downregulated [*L. mex* PPs (↓)] phosphoproteins in (**A**) log phase promastigote (LPP) and (**B**) axenic amastigote (AXA) life cycle stages. (**C** and **D**) Most enriched InterPro domains in the upregulated and downregulated *L. mexicana* RNA-binding phosphoproteins, respectively, in the LPP. (**E** and **F**) Most enriched InterPro domains in the upregulated and downregulated *L. mexicana* RNA-binding phosphoproteins, respectively, in the AXA.

In the LPP, 169 out of 623 downregulated phosphoproteins and 76 out of 236 upregulated phosphoproteins were found to be RBPs that exhibited a decreased RNA-binding capacity upon treatment with tanespimycin (Fig. 6A). Similarly, in the AXA, 162 out of 430 downregulated phosphoproteins and 182 out of 614 upregulated phosphoproteins were also found to be RBPs with a decreased RNA-binding capacity upon treatment with tanespimycin (Fig. 6B). In the LPP, the upregulated (Fig. 6C) and downregulated (Fig. 6D) RNA-binding phosphoproteins showed alpha tubulin and 40S ribosomal protein S1/3, respectively, among the most enriched InterPro domains. In contrast, comparison of interprotein domain occurrences in the RNA-binding phosphoproteins of the AXA revealed that RNA helicase and heat shock protein HSP90 were among the most enriched InterPro domains in the upregulated (Fig. 6E) and downregulated (Fig. 6F) phosphoproteins, respectively. The HSP90 InterPro domain was found in the proteins E9B3L2 (LmxM.32.0316, LmxM.32.0312 and LmxM.32.0314), E9AM02 (LmxM.08_29.0760), and E9B478 (LmxM.32.2390).The increased phosphorylation detected in RNA helicases such as the ATP-dependent RNA helicase LmxM.28.1310 upon HSP90 inhibition agrees with previous reports suggesting involvement of the HSP90 in RNA metabolism such as RNA splicing, RNA transport, and RNA decay (19 – 21).

**Fig 7:**
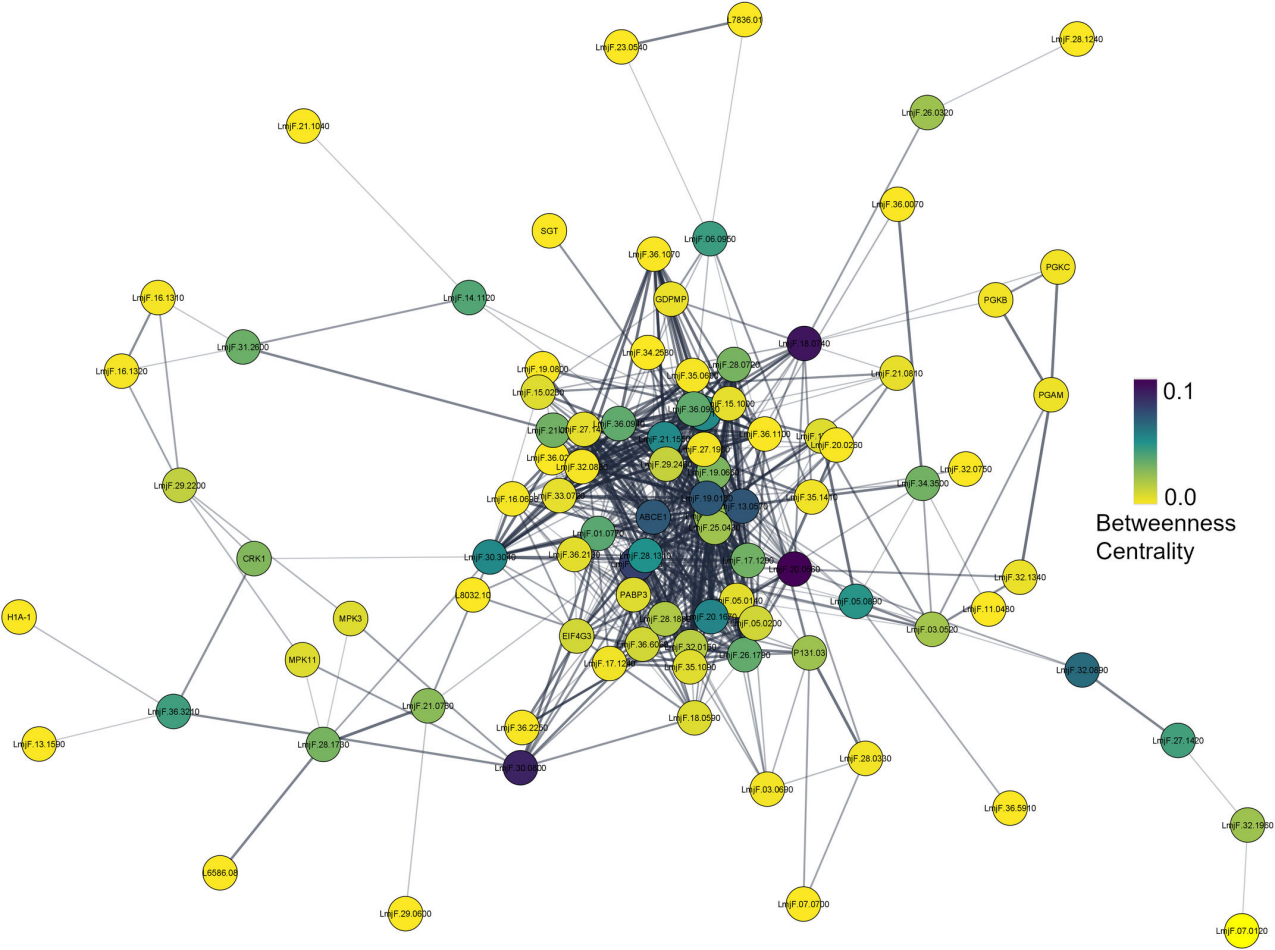
Protein-protein interaction network of RNA-binding phosphoproteins upregulated during HSP90 inhibition in *L. mexicana* axenic amastigotes (AXAs) constructed using publicly available STRING database of *L. major* Friedlin strain. The nodes are colored according to their betweenness centrality in the network.

Next, we constructed protein-protein interaction (PPI) networks of the HSP90 inhibition-perturbed RNA-binding phosphoproteins. Network analysis revealed the important node properties of degree centrality, betweenness centrality, and closeness centrality in the four PPI networks (Table S8). In the AXA, the PPI network of the upregulated RNA-binding phosphoproteins revealed cytidine triphosphate synthase (LmjF.20.0560), elongation factor Tu (LmjF.18.0740), Rac serine-threonine kinase (LmjF.30.0800), and ATP-dependent DEAD-box helicase (LmjF.28.1530) as crucial nodes with the highest betweenness centrality (Fig. 7). In contrast, the network of the downregulated AXA phosphoproteins revealed elongation factor-1 gamma (LmjF.09.0970) and beta tubulin (LmjF.33.0794) with the highest betweenness centrality (Fig. S13). In the LPP downregulated PPI network, actine-like protein and chaperonin alpha subunit were identified with the highest betweenness centrality (Fig. S14). In the LPP upregulated PPI network, several nodes were identified with high betweenness centrality (Fig. S15). These include glucose-related protein 78 (LmjF.28.1200), elongation factor 2 (LmjF.36.0180), nucleolar protein (LmjF.10.0210), protein kinase A (LmjF.13.0160), stress-induced protein STI1 (LmjF.08.1110), 14-3-3 protein (LmjF.11.0350), and chaperonin TCP20 (LmjF.13.1660). Interestingly, the high betweenness centrality node, Rac serine-threonine kinase (LmjF.30.0800), in the AXA upregulated phosphoproteins was identified as a crucial node of information flow in the LPP upregulated phosphoproteins as well, suggesting a potential role of phosphorylation of this PK in regulating the HSP90 inhibition stress in both life cycle stages of *Leishmania*. In contrast, the cytidine triphosphate synthase (LmjF.20.0560) and the ATP-dependent DEAD-box helicase (LmjF.28.1530) were unique high-betweenness centrality nodes of the AXA phosphoproteome (Fig. 7), suggesting their phosphorylation playing a role in the life cycle-specific regulation of the HSP90 inhibition.

## DISCUSSION

The combination of phosphoproteome enrichment with TMT labeling-based quantitative proteomic MS enabled comprehensive profiling of quantitative changes in *L. mexicana* phosphoproteome across its three life cycle stages. Previous studies based on gel-based quantitative methods have shown an increased phosphorylation of HSP90 and its cochaperone HSP70 during the differentiation of *Leishmania* from promastigote to amastigote (6). Our quantitative proteomic MS study not only confirmed these earlier findings but more importantly provided relative changes in the phosphorylation levels of thousands of *Leishmania* proteins during the differentiation. These include many essential PKs, PPs, hydrolases, RBPs, and helicases. We have shown that while phosphorylation of chaperone proteins is upregulated in the *L. mexicana* amastigotes, PK phosphorylation was slightly more prominent in the promastigotes. Many PKs are regulated by phosphorylation events (22, 23). Phosphorylation of the activation loop of PKs can increase the enzymatic activity by promoting a conformational change that allows the kinase to bind and phosphorylate its substrates more efficiently (22). Conversely, phosphorylation of inhibitory sites can reduce the kinase activity by preventing the activation loop from adopting its catalytically active conformation (22). Thus, the biological effects of phosphorylation of PKs are context dependent and mediated by a network of protein interactions.

Our data accurately captured upregulation of phosphorylation of a set of PKs in the *L. mexicana*a mastigote. MEKK-related kinase 1 (MRK1, LmxM.31.0120) was the most highly upregulated PK in the AXA. In higher eukaryotes, MEKK1 serves an important regulator of ERK, JNK, and MAPK pathways, and when activated by stresses that alter cytoskeleton and cell shape, the MEKK1 signals to protect the cell from apoptosis (24). More importantly, MRK1, MPK10, MPK15, and PKAC3 from the upregulated phosphorylated kinases in the AXA were recently identified as essential for successful differentiation of *L. mexicana* promastigotes to amastigote and survival in both macrophages and in mouse (18). Similarly, the AGC essential kinase-1 (AEK1), another PK with upregulated phosphorylation in the AXA, was reported as an essential PK in the *L. mexicana* promastigotes (18). Further investigations are required to establish the specific mechanisms and functional consequences of the increased phosphorylation detected in the essential PKs in the AXA. Understanding the exact significance of phosphorylation of these PKs in the parasite’s life cycle and survival in the host cells may be crucial in the development of potential therapeutic interventions.

Protein size and isoelectric point are crucial factors that can influence protein solubility and interactions with other proteins and other biomolecules such as nucleic acids, lipids and metabolites, and can affect the efficiency and specificity of these interactions and signaling pathways (25). Smaller proteins may be more prone to dynamic interactions with other proteins, and proteins with more acidic isoelectric points tend to have a greater propensity for interactions with positively charged proteins. Therefore, the observed differences in protein size and isoelectric points between phosphorylation substrates in the amastigotes and the promastigotes indicate that there are distinct regulatory networks or signaling pathways that operate at each stage, and these networks may be optimized for different types of protein-protein interactions or cellular responses. It is also possible that these differences reflect changes in the cellular environment or in the expression of specific PKs and PPs that regulate protein phosphorylation.

Our quantitative proteomic data captured global changes in the phosphorylation landscape upon HSP90 inhibition in the LPP, SPP, and AXA life cycle stages of *L. mexicana*. The HSP90 inhibition-induced perturbations in the global phosphorylation network indicate interplay between HSP90 and protein kinome and phosphatase signaling pathways in the organism. Although the interactions and client proteins of HSP90 in *Leishmania* are poorly known, many client proteins of HSP90 in higher eukaryotes are PKs and PPs (26 – 30). These client proteins depend on HSP90 for proper folding and stabilization. Also, by modulating the activity of kinases and phosphatases, HSP90 can affect various signaling cascades they are involved in. When a client kinase is activated, HSP90 may assist in maintaining its active conformation and protect it from degradation. On the other hand, inhibition of HSP90 can lead to degradation of client kinases or alteration of their subcellular localization, which could interfere with the activity and dynamics of their entire signaling cascades.

It is the amastigote form that causes leishmaniasis in vertebrate hosts. Therefore, identification of druggable targets and pathways in the amastigote is crucial. The increased phosphorylation of specific PKs detected in the amastigote following HSP90 inhibition suggests an increased reliance of the parasite in the amastigote stage on these PKs to mitigate the detrimental effects of the HSP90 inhibition stress. These include kinases with potential therapeutic targeting scope such as the mitogen-activated PKs MPK3, MPK10, MPK11, MPK12, and MPK14; cell division PKs CRK1, CRK3, and CRK9; and glycogen synthase kinase GSK-3β, AEK1, and MRK1 (18, 31, 32). This suggests that investigating targeting of these PKs either alone or in combination with HSP90 inhibition against leishmaniasis may represent valuable areas for drug discovery. Collectively, the findings of this study offer a plethora of valuable insights that can be utilized to explore the molecular mechanisms and implications of inhibiting HSP90 in the *Leishmania* parasite in future research.

## MATERIALS AND METHODS

### *Leishmania mexicana* culture

*L. mexicana* M379 strain (MNYC/BC/62 /M379) promastigotes from frozen stock were quickly defrosted in a water bath at 37°C and inoculated in 10 mL Schneider’s insect medium (Sigma-Aldrich) in T-25 flasks supplemented with 0.4-g/L NaHCO_3_, 0.6-g/L CaCl_2_, and 15% heat-inactivated fetal bovine serum (FBS; Thermo Fisher Scientific, South American origin) at pH 7.0. The parasites were incubated at 26°C for 1–2 days. The parasites were in log phase by this stage with many dividing cells. This culture was used to inoculate another culture by seeding 5 × 10^5^ parasites/mL in 60 mL complete Schneider’s insect medium and incubated at 26°C for 2–3 days. In order to generate SPPs, the incubation was continued for 8–9 days. AXAs were generated starting from fresh LPP cultures using changes in pH and temperature of the culture medium as described earlier (14). Briefly, the *L. mexicana* promastigotes in log phase on day 3 of the culture were transferred to 60 mL of pH 5.5 Schneider’s insect medium supplemented with 20% heat-inactivated FBS and incubation continued at 26°C. On days 8 and 9, the parasites were in stationary phase and were transferred to 60 mL of pH 5.5 Schneider’s insect medium supplemented with 20% heat-inactivated FBS and incubated at 32°C. On days 11 and 12, the parasites were completely differentiated into amastigote stage. The growth and morphology of parasites were observed under an optical microscope, and parasite numbers in cultures at all stages were measured using a hemocytometer.

### Tanespimycin treatment and cell lysate preparation

*L. mexicana* parasites in LPP, SPP, and AXA life cycle stages were treated with 1 µM tanespimycin (Selleckchem, 10-mM stock solution in DMSO) or DMSO (control, 3 µM) in fresh complete Schneider’s insect medium in T-75 flasks (30 mL culture medium per flask, cell density of 5 × 10^6^ parasites/mL) for a total duration of 16 h in biological triplicates. Following treatments, the parasites were washed three times with ice-cold phosphate buffered saline (PBS) and lysed immediately in ice-cold lysis buffer (20 mM Tris-HCl, pH 8.5, 8 M urea, phosphatase inhibitors [PhosSTOP, Roche], 2 mM dithiothreitol [DTT], and 1 mM phenylmethylsulfonyl fluoride [PMSF]) by passing the parasites through 29G needles 8–10 times. Lysate debris were eliminated by centrifugation at 16,000 × *g* for 10 min at 4°C. After centrifugation, the protein samples were reduced by DTT treatment (10 mM, 60 min, 35°C) and alkylated by iodoacetamide (20 mM, 45 min at room temperature in the dark). The samples were digested overnight at 37°C with sequencing-grade modified trypsin (Promega) at an enzyme to protein ratio of 1:40. The samples were then acidified with trifluoroacetic acid (TFA) (0.1% [vol/vol] final concentration, Sigma-Aldrich), centrifuged at 16,000 × *g* for 10 min and the supernatant was collected. The tryptic peptides were then desalted on C-18 Sep-Pak Classic cartridges (Waters, WAT051910) following manufacturer’s instructions. The peptides were evaporated to complete dryness in a speed vacuum concentrator and stored at −80°C until required.

### Phosphopeptide enrichment

Phosphopeptides were enriched using High-Select Fe-NTA Phosphopeptide Enrichment Kit (Thermo Scientific) following manufacturer’s protocols. The eluted phosphopeptides were dried immediately in a speed vacuum concentrator. The samples were then acidified with 0.1% TFA and desalted on C-18 Sep-Pak Classic cartridges (Waters, WAT051910) following manufacturer’s instructions. The peptides were evaporated to complete dryness in a speed vacuum concentrator and subjected to TMT labeling.

### TMT labeling

TMT labeling of the desalted phosphopeptides was carried out using TMTsixplex isobaric label reagent set (Thermo Fisher Scientific). The phosphopeptides of each experimental condition were dissolved in 100 µL of 100 mM triethylammonium bicarbonate and treated with room temperature equilibrated and freshly dissolved unique TMT label reagent in 41 µL anhydrous acetonitrile. The labeling reactions were run for 1 h at room temperature, following which 10 µL of 5% solution of hydroxylamine was added and incubated for 15 min at room temperature to quench the reactions. All experiments were performed in biological triplicates. A total of 18 samples were generated across the three life cycle stages, with six samples corresponding to each life cycle stage. In the six samples generated per life cycle stage, three were phosphopeptides enriched without tanespimycin treatment (dimethyl sulfoxide [DMSO] control, three replicates), and the remaining three samples were phosphopeptides enriched with tanespimycin treatment (three replicates). These six samples of each life cycle stage were then combined together and concentrated to complete dryness in a speed vacuum concentrator. Thus, three separate TMT labeled master mix samples were generated across the three life cycle stages.The samples were then redissolved in 0.1% TFA, desalted, and cleaned-up using Pierce Peptide Desalting Spin Columns (Thermo Fisher Scientific) following the manufacturer’s instructions. The samples were dried in a speed vacuum concentrator and stored at −80°C until required.

### Nano LC-MS/MS data acquisition

The liquid chromatrography-tandem mass spectrometry (LC-MS/MS) analyses of TMT-labeled peptides were performed on an Orbitrap Fusion Lumos Mass Spectrometer (Thermo Fisher Scientific) coupled with a Thermo Scientific Ultimate 3000 RSLCnano UHPLC system (Thermo Fisher Scientific). Desalted and TMT-labeled tryptic peptides dissolved in 0.1% formic acid (FA) were first loaded onto an Acclaim PepMap 100 C18 trap column (5-µm particle size, 100-µm id × 20 mm, TF164564) heated to 45°C using 0.1% FA/H_2_O with a flow rate of 10 µL/min, then separated on an Acclaim PepMap 100 NanoViper C18 column (2-µm particle size, 75 µm id × 50 cm, TF164942) with a 5%–38% acetonitrile (ACN) gradient in 0.1% FA over 125 min at a flow rate of 300 nL/min. The full MS spectra (*m*/*z* 375–1,500) were acquired in Orbitrap at 120,000 resolution with an AGC target value of 4e^5^ for a maximum injection time of 50 ms. High-resolution HCD MS2 spectra were generated in positive ion mode using a normalized collision energy of 38% within a 0.7 *m*/*z* isolation window using quadrupole isolation. The AGC target value was set to 10e^4^, and the dynamic exclusion was set to 45 s. The MS2 spectra were acquired in Orbitrap with a maximum injection time of 54 ms at a resolution of 30,000 with an instrument determined scan range beginning at *m*/*z* 100. To ensure quality peptide fragmentation, a number of filters were utilized, including peptide monoisotopic precursor selection, minimum intensity exclusion of 10e^3^, and exclusion of precursor ions with unassigned charge state as well as charge state of +1 or superior to +7 from fragmentation selection. To prevent repeat sampling, a dynamic exclusion with exclusion count of 1, exclusion duration of 30 s, mass tolerance window of ±7 ppm, and isotope exclusion were used.

### Proteome data processing and analysis

All raw LC-MS/MS data were processed using MaxQuant software (33) (version 1.6.3.4) with integrated Andromeda database search engine (34). The MS/MS spectra were queried against *L. mexicana* sequences from UniProt KB (8,559 sequences, UniProt Taxonomy ID: 5665). The following search parameters were used: reporter ion MS2 with multiplicity 6plex TMT, trypsin digestion with maximum two missed cleavages, carbamidomethylation of cysteine as a fixed modification, oxidation of methionine, acetylation of protein N-termini and phosphorylation of serine, threonine and tyrosine residues as variable modifications, minimum peptide length of 6, maximum number of modifications per peptide set at 5, and protein false discovery rate (FDR) 0.01. Appropriate correction factors for the individual TMT channels for both lysine side-chain labeling and peptide N-terminal labeling as per the TMT-6plex kits used (Thermo Fisher Scientific) were configured into the database search. The proteinGroups.txt files from the MaxQuant search outputs were processed using Perseus software (35) (version 1.6.2.3). Sequences only identified by site, reverse sequences, and potential contaminants were filtered out. A requirement of six nonzero valid value were set across the eighteen reporter intensity corrected main columns of the three life cycle stages.The reporter intensities were normalized by Z-score and transformed to log2 scale. Proteins identified with fewer than two unique peptides were discarded and a modified *t*test with permutation-based FDR statistics (250 permutations) was applied to compare the different life cycle stages and tanespimycin-treated and nontreated groups.

### Bioinformatic analysis

Gene ontology (GO) terms (molecular function, biological process, and cellular component) of the phosphoprotein data sets were derived from TriTrypDB (tritrypdb.org) (36). Custom R scripts with R 64-bit (version 4.2.3) along with R package ggplot2 (version 3.4.2) were used for visualizing the GO terms. InterPro domain occurrences in the phosphoproteins were derived from bioinformatics analysis of the protein IDs using the Database for Annotation, Visualization and Integrated Discovery (DAVID) bioinformatics resources (version 6.8) (37). Grand average hydrophobicity values of the phosphoproteins and the entire *L. mexicana* proteome were calculated using the ExPASy (38) tool ProtParam. Isoelectric points and molecular weights were computed using the ExPASy tool Compute pI/Mw. Cumulative distributions of physicochemical properties were derived using custom R scripts and visualized using the R package ggplot2. Protein-protein interaction network analyses were performed by using the publicly available STRING database (version 11.5) (39)of *L. major* strain Friedlin. The open-source software platform Cytoscape (version 3.9.1) (40) was used for refining, analyzing, and visualizing the protein interaction network.

## Data Availability

All raw mass spectrometry proteomics data have been deposited to the ProteomeXchange Consortium via the PRIDE partner repository with the data set identifier PXD043002.

## SUPPLEMENTAL MATERIAL

Supplemental figures spectrum.02960-23-s0001.pdf Fig. S1 to S15.

Table S1 spectrum.02960-23-s0002.xlsx Phosphoproteins of L. mexicana axenic amastigote, log phase promastigote and stationary phase promastigote life cycle stages.

Table S2 spectrum.02960-23-s0003.xlsx Stage specific variations in the phosphoproteome of L. mexicana (axenic amastigote vs log phase promastigote).

Table S3 spectrum.02960-23-s0004.xlsx Stage specific variations in the phosphoproteome of L. mexicana (axenic amastigote vs stationary phase promastigote).

Table S4 spectrum.02960-23-s0005.xlsx Stage specific variations in the phosphoproteome of L. mexicana (stationary phase promastigote vs log phase promastigote).

Table S5 spectrum.02960-23-s0006.xlsx Effect of HSP90 inhibition via tanespimycin treatment on the phosphoproteome of L. mexicana log phase promastigotes (LPPs).

Table S6 spectrum.02960-23-s0007.xlsx Effect of HSP90 inhibition via tanespimycin treatment on the phosphoproteome of L. mexicana stationary phase promastigotes (SPPs).

Table S7 spectrum.02960-23-s0008.xlsx Effect of HSP90 inhibition via tanespimycin treatment on the phosphoproteome of L. mexicana axenic amastigotes (AXAs).

Table S8 spectrum.02960-23-s0009.xlsx Protein-protein interaction network parameters of perturbed RNA-binding phosphoproteins upon HSP90 inhibition.

ASM does not own the copyrights to Supplemental Material that may be linked to, or accessed through, an article. The authors have granted ASM a non-exclusive, world-wide license to publish the Supplemental Material files. Please contact the corresponding author directly for reuse.
