# Supplementary material for: Inhibition of HSP90 distinctively modulates the global phosphoproteome of Leishmania mexicana developmental stages
Source: Microbiol Spectr. 2023 Oct 31;11(6):e02960-23. doi: 10.1128/spectrum.02960-23 (PMC10715028; doi:10.1128/spectrum.02960-23)
Supplement: Supplemental figures — Fig. S1 to S15. [file spectrum.02960-23-s0001.pdf]

# **Inhibition of HSP90 distinctively modulates the global phosphoproteome of *Leishmania mexicana* developmental stages**

Exequiel O. J. Porta,[a] Liqian Gao,[b] Paul W. Denny,[c] Patrick G. Steel,[a] Karunakaran Kalesh[d,e,*]

[a]Department of Chemistry, Durham University, Durham, United Kingdom

[b]School of Pharmaceutical Sciences, Shenzhen Campus of Sun Yat-sen University, Shenzhen, China

[c]Department of Biosciences, Durham University, Durham, United Kingdom

[d]School of Health and Life Sciences, Teesside University, Middlesbrough, United Kingdom

[e]National Horizons Centre, Darlington, United Kingdom

*Address correspondence to Karunakaran Kalesh, k.karunakaran@tees.ac.uk

MTETFAFQAEINQLMSLIINTFYSNKEIFLRELISNAS$_{38}$DACDKIRYQS$_{48}$LTDPSVLGDATRLCVRVVPDKENKTLTV
EDNGIGMTKADLVNNLGTIARSGTKAFMEALEAGGDMSMIGQFGVGFYSAYLVADRVTVTSKNNSDEVYVWESS
AGGTFTITSAPESDMKRGTRITLHLKEDQLEYLEVRRLKELIKKHSEFIGYDIELMVEKTT$_{211}$EKEVT$_{216}$DEDEEEAKK
ADEDGEEPKVEEVT$_{239}$EGEEGKKKKTKKVKEVT$_{256}$KEYEVQNKHKPLWTRDPKDVTKEEYAAFYKAIS$_{289}$NDWE
DPAATKHFSVEGQLEFRSIMFVPKRAPFDMFEPNKKRNNIKLYVRRVFIMDNCEDLCPDWLGFVKGVVDSEDLPLN
IS$_{371}$RENLQQNKILKVIRKNIVKKCLEMFEEVAENKEDYKQFYEQFGKNIKLGIHEDTANRKKLMELLRFYSTESGEE
MTTLKDYVTRMKAEQKSIYYITGDSKKKLESS$_{477}$PFIEQAKRRGFEVLFMTEPIDEYVMQQVKDFEDKKFACLTKEG
VHFEES$_{526}$EEEKRQREEEKAACEKLCKTMKEVLGDKVEKVTVSERLSTSPCILVTSEFGWSAHMEQIMRNQALRDS$_{594}$S$_{595}$MAQYMMSKKTMELNPKHPIIKELRRRVEADENDKAVKDLVFLLFDTSLLTSGFQLEDPTGYAERINRMIKLG
LSLDEEEEEAVEAAVAETAPAEVTAGTSSMEQVD

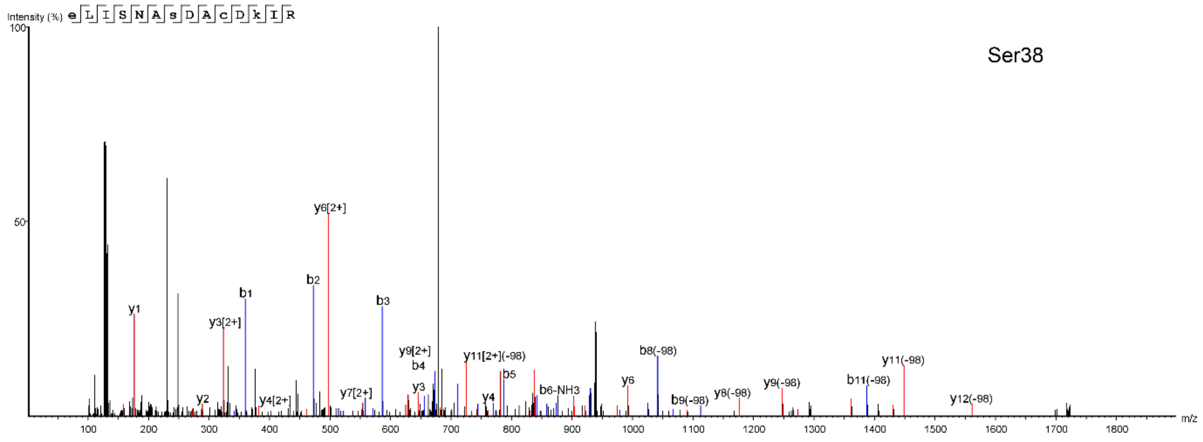


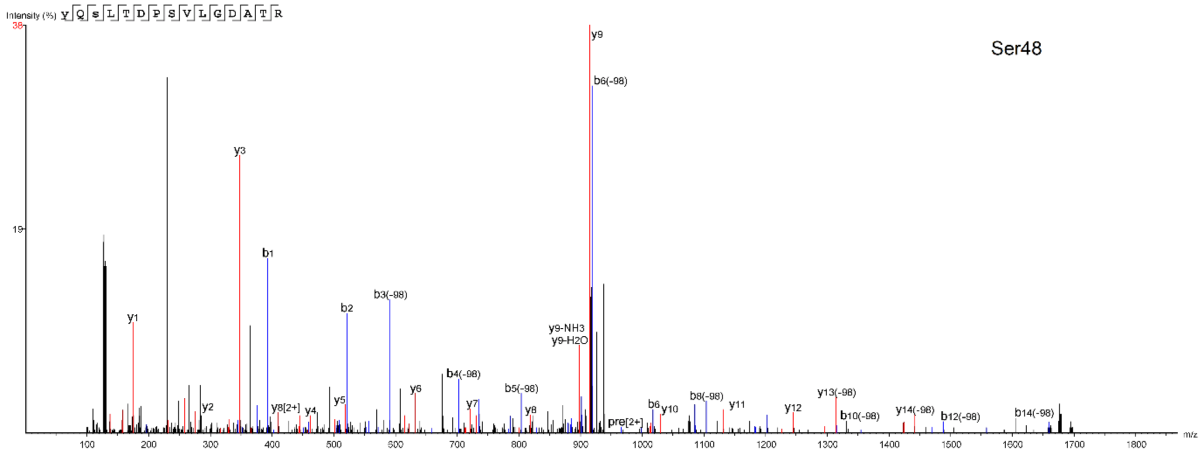

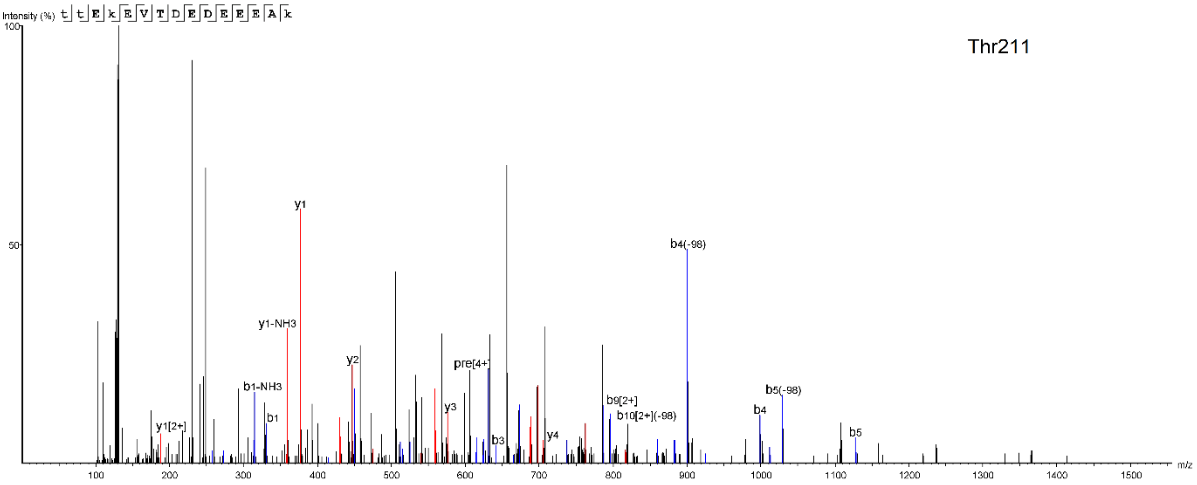
Intensity (%)
t t E k E V T D E D E E E A k
Thr211
y1
y1-NH3
b4(-98)
y2
pre[4+]
b1-NH3
b1
y1[2+]
y3
b3
y4
b9[2+]
b10[2+](-98)
b4
b5(-98)
b5
m/z
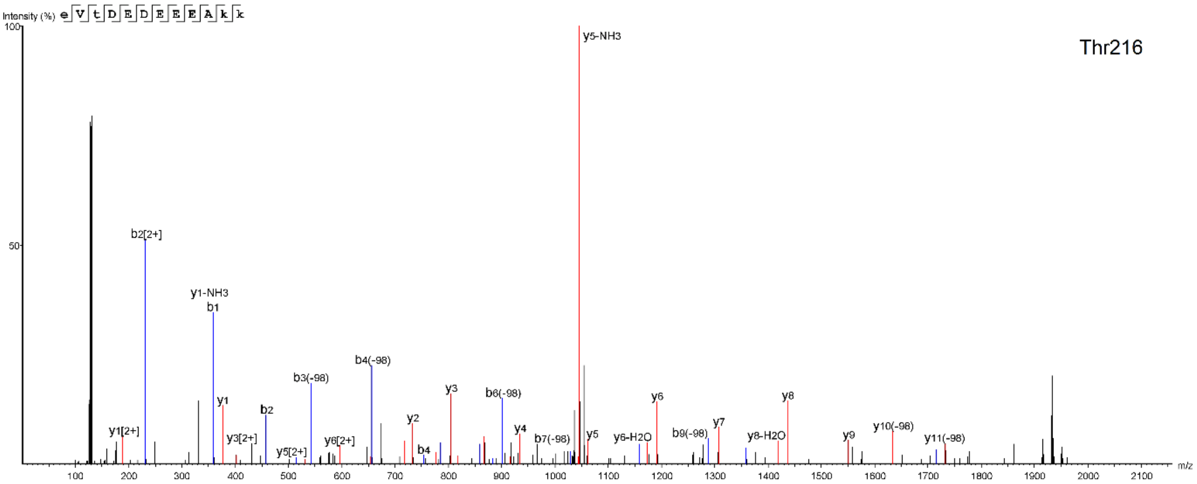
Intensity (%)
e V t D E D E E E A k k
Thr216
y5-NH3
b2[2+]
y1-NH3
b1
b4(-98)
b3(-98)
y3
b6(-98)
y6
y8
y1
b2
y1[2+]
y3[2+]
y5[2+]
y6[2+]
y2
b4
y4
b7(-98)
y5
y6-H2O
b9(-98)
y7
y8-H2O
y9
y10(-98)
y11(-98)
m/z
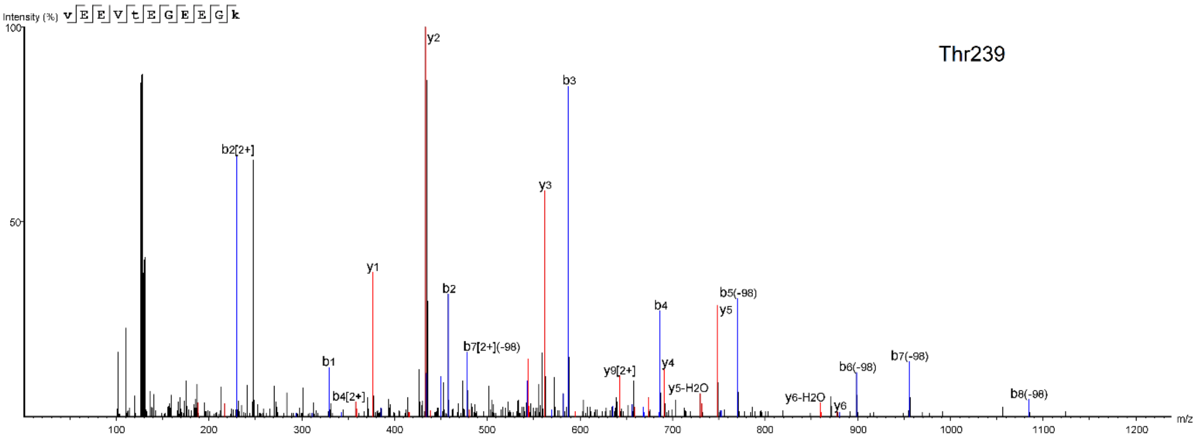
Intensity (%)
v E E V t E G E E G k
Thr239
y2
b3
b2[2+]
y3
y1
b2
b1
b4[2+]
b7[2+](-98)
b4
b5(-98)
y5
y9[2+]
y4
y5-H2O
y6-H2O
y6
b6(-98)
b7(-98)
b8(-98)
m/z

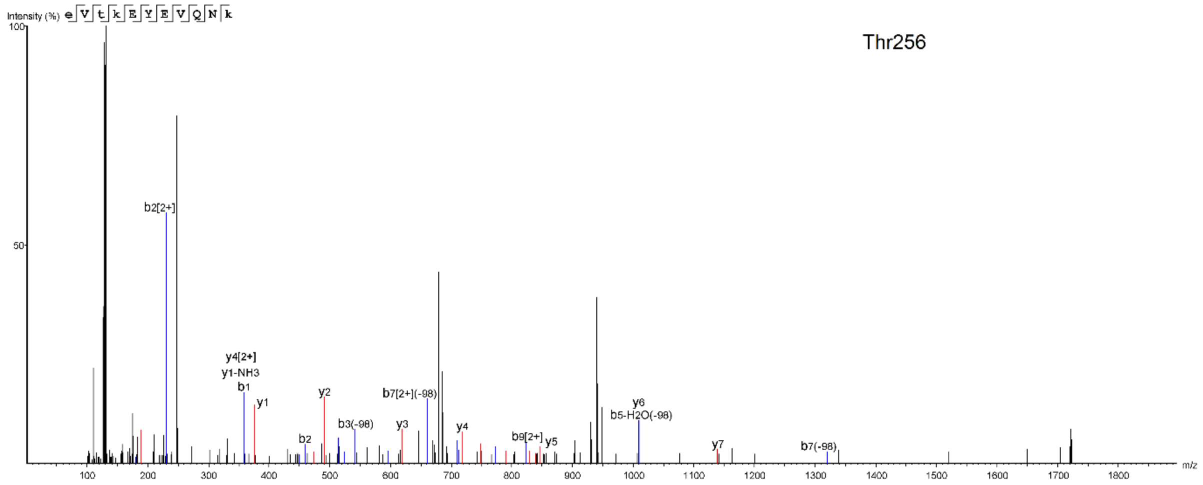

Intensity (%) e V t k E Y E V Q N k
Thr256
b2[2+]
y4[2+]
y1-NH3
b1
y1
y2
b2
b3(-98)
y3
b7[2+](-98)
y4
b9[2+]
y5
y6
b5-H2O(-98)
y7
b7(-98)
m/z


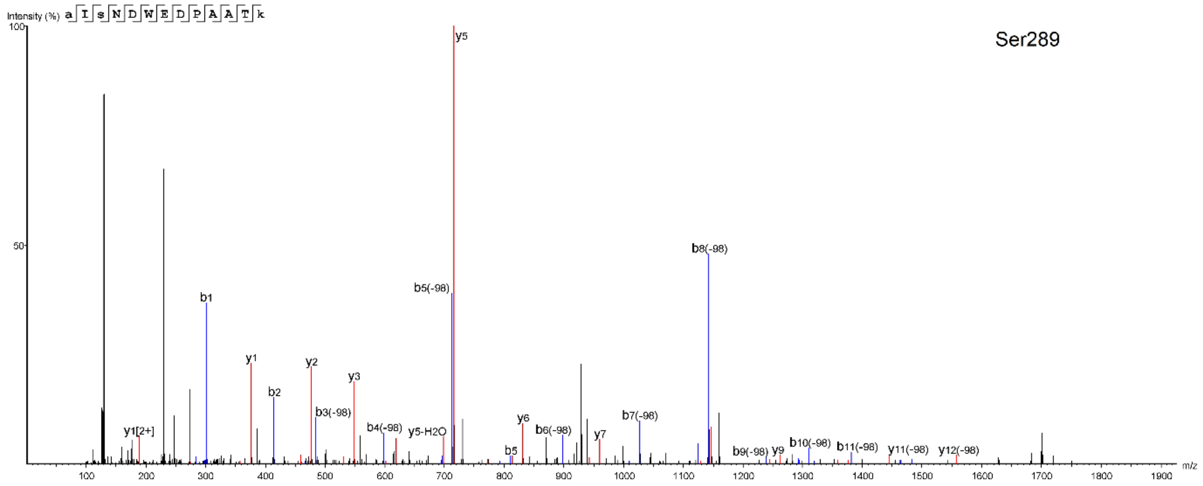

Intensity (%) a I s N D W E D P A A T k
Ser289
y5
b8(-98)
b5(-98)
b1
y1
y2
y3
b2
b3(-98)
y1[2+]
b4(-98)
y5-H2O
y6
b5
b6(-98)
y7
b7(-98)
b9(-98)
y9
b10(-98)
b11(-98)
y11(-98)
y12(-98)
m/z


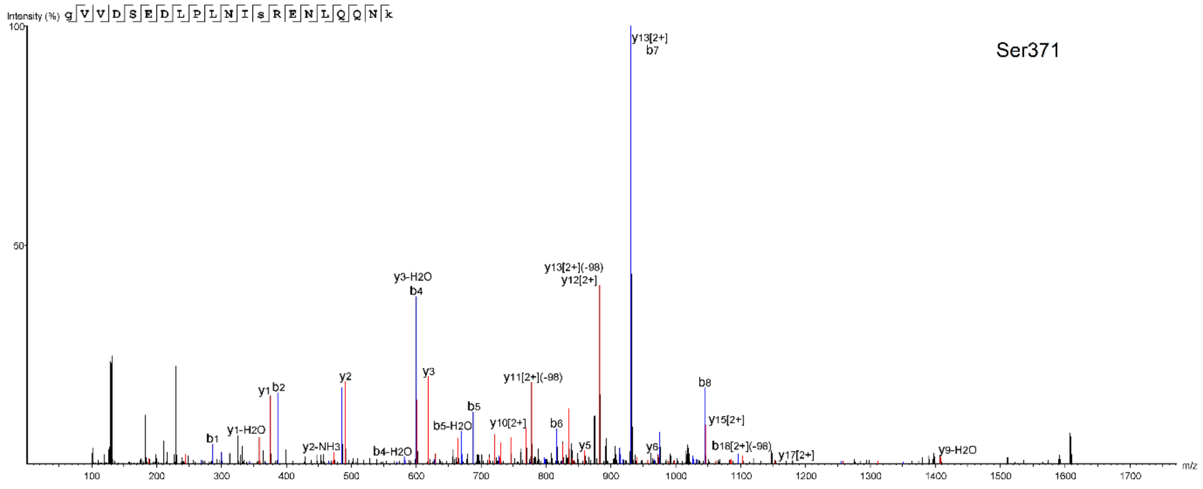

Intensity (%) g V V D S E D L P L N I s R E N L Q Q N k
Ser371
y13[2+]
b7
y13[2+](-98)
y12[2+]
y3-H2O
b4
y3
y11[2+](-98)
y2
y1
b2
b1
y1-H2O
y2-NH3
b4-H2O
b5
b5-H2O
y10[2+]
b6
y5
y6
b8
y15[2+]
b18[2+](-98)
y17[2+]
y9-H2O
m/z

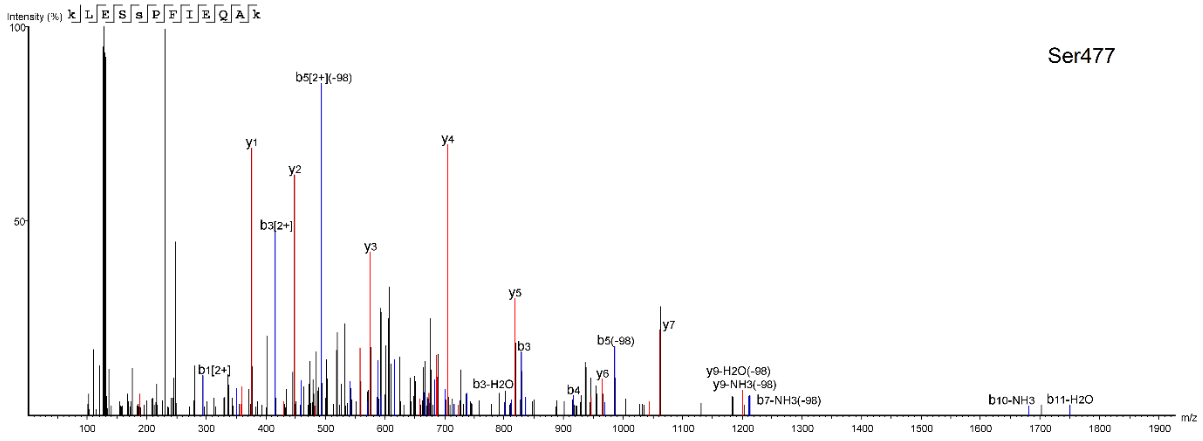
Intensity (%)
k L E S s P F I E Q A k
Ser477
b5[2+](-98)
y1
y4
y2
b3[2+]
y3
y5
b1[2+]
b3
b3-H2O
b4
b5(-98)
y6
y7
y9-H2O(-98)
y9-NH3(-98)
b7-NH3(-98)
b10-NH3
b11-H2O
m/z

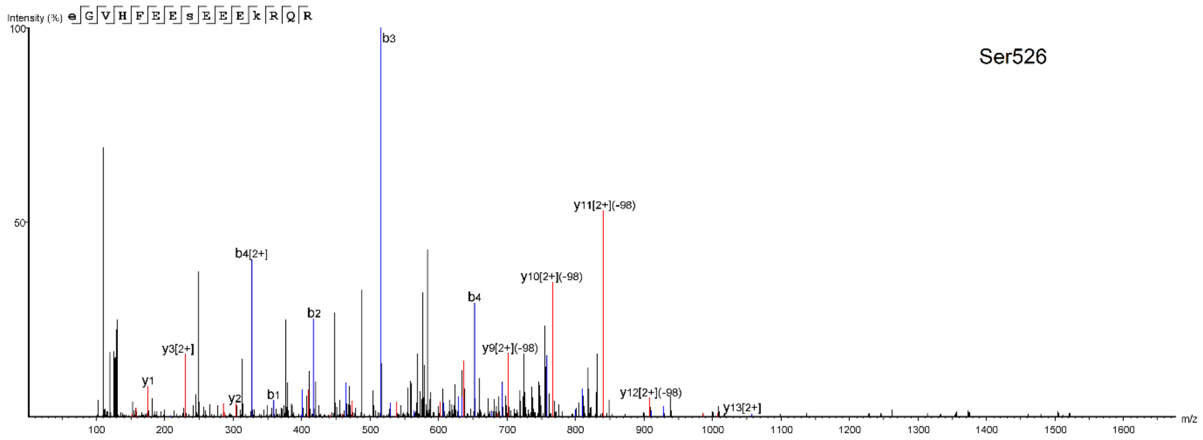
Intensity (%)
e G V H F E E s E E E k R Q R
Ser526
b3
y11[2+](-98)
b4[2+]
y10[2+](-98)
b4
b2
y3[2+]
y9[2+](-98)
y1
y2
b1
y12[2+](-98)
y13[2+]
m/z

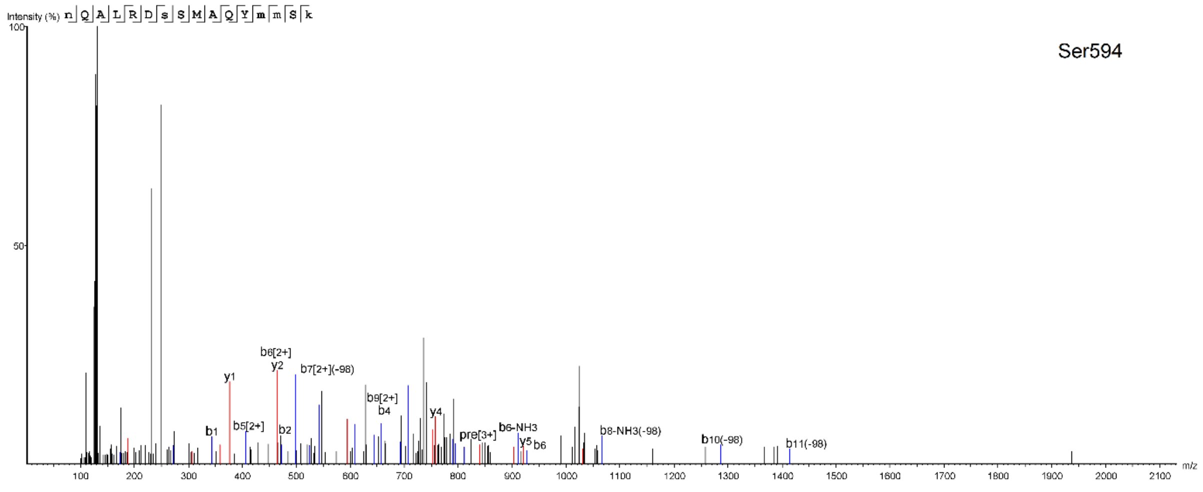
Intensity (%)
n Q A L R D s S M A Q Y m m S k
Ser594
b6[2+]
y2
b7[2+](-98)
y1
b9[2+]
b4
y4
b5[2+]
b1
b2
pre[3+]
b6-NH3
y5
b6
b8-NH3(-98)
b10(-98)
b11(-98)
m/z

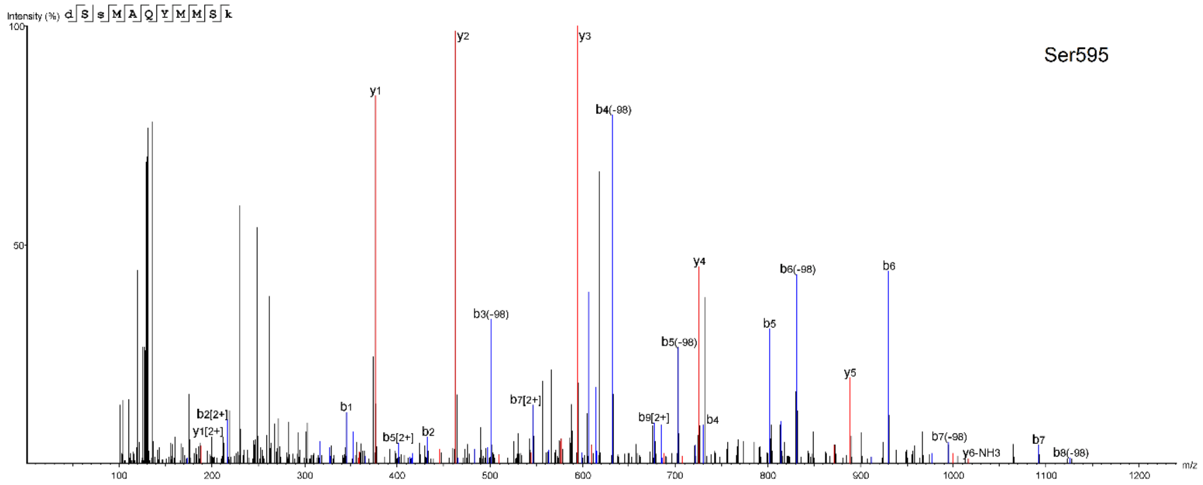


**FIG. S1** MS/MS spectra of phosphorylated tryptic peptides from *L. mexicana* HSP90.

Mitogen-activated protein kinase 2 (MPK2; LmxM.36.0720)

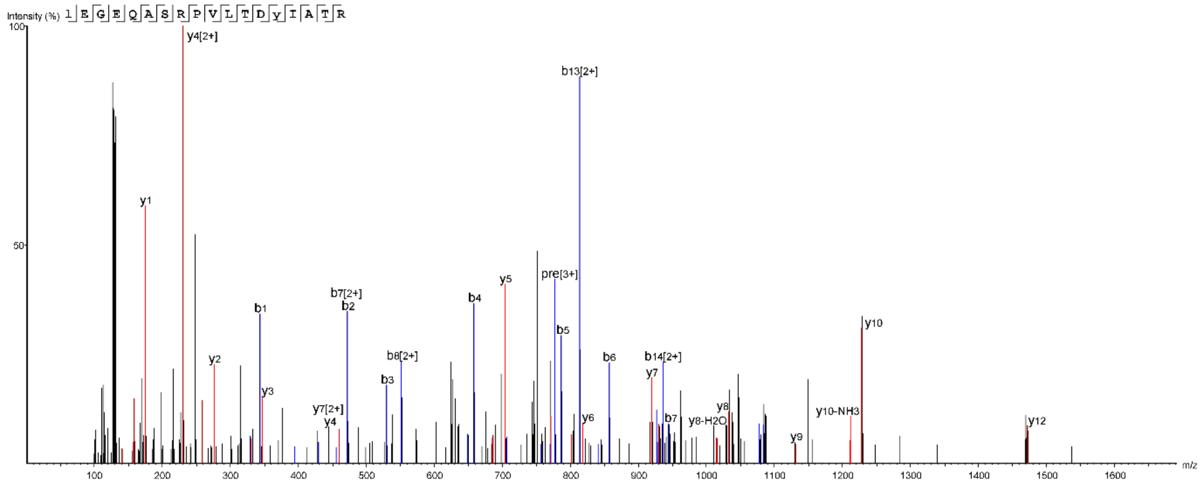


Mitogen-activated protein kinase 3 (MPK3; LmxM.10.0490)

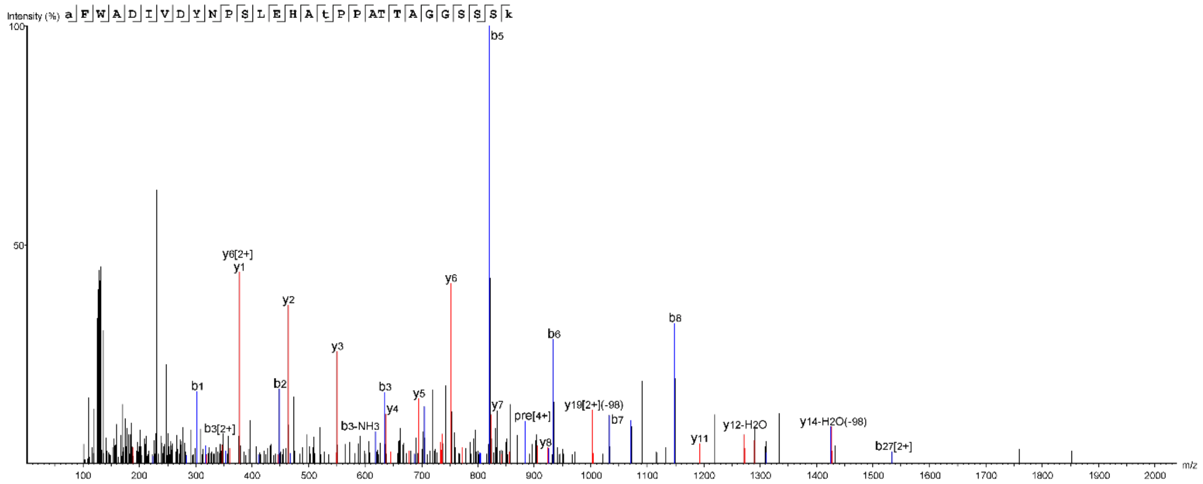

## Mitogen-activated protein kinase 5 (MPK5; LmxM.29.2910)

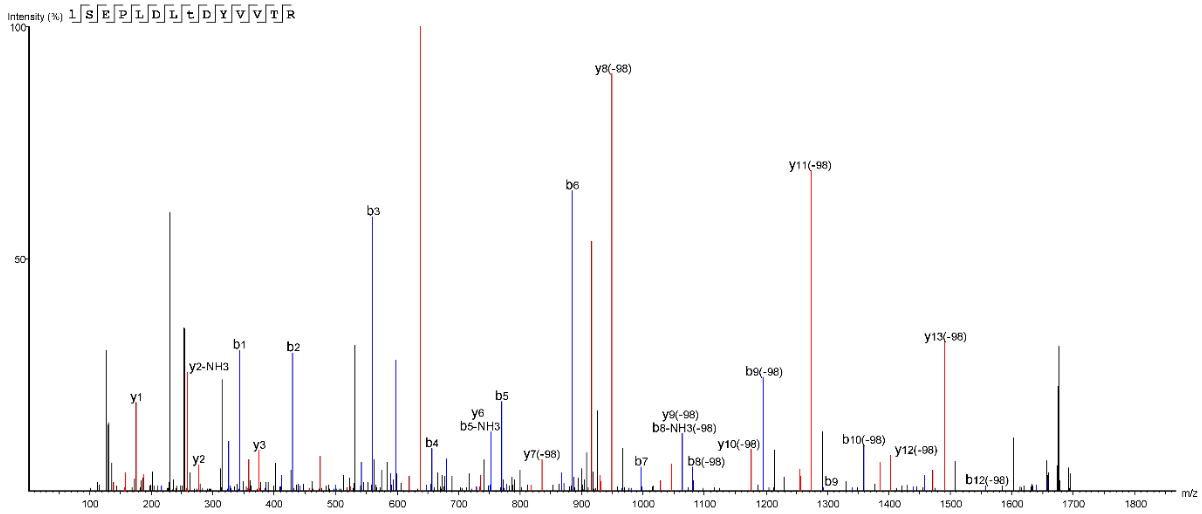


## Mitogen-activated protein kinase 6 (MPK6; LmxM.31.3250)

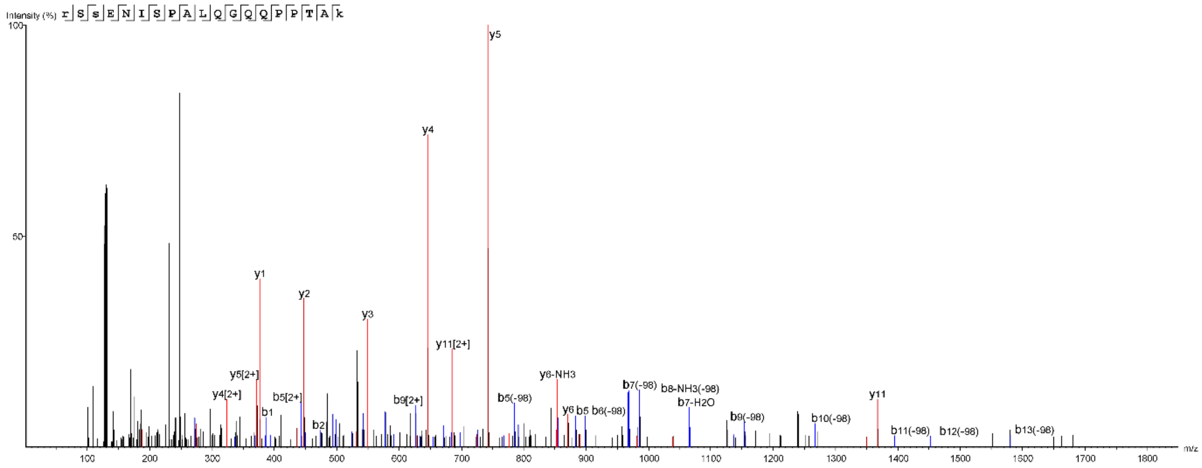


## Mitogen-activated protein kinase 9 (MPK9; LmxM.19.0180)

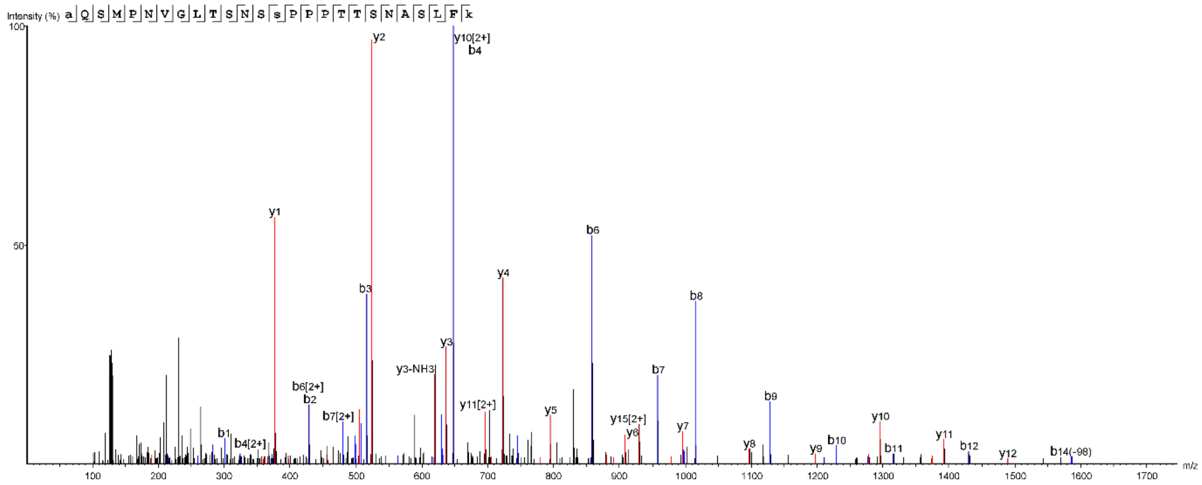

## Mitogen-activated protein kinase 11 (MPK11; LmxM.32.1380)

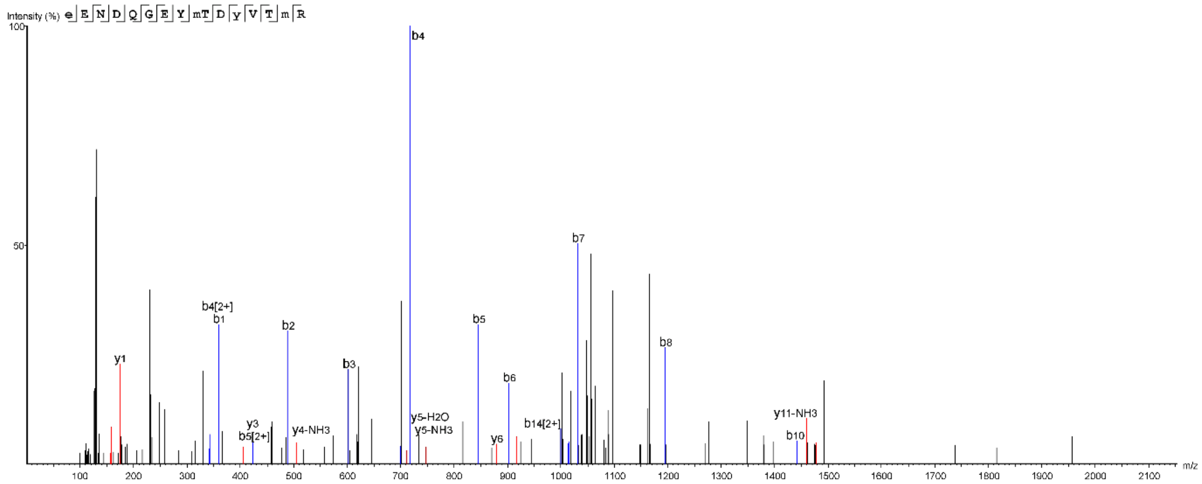


## Mitogen-activated protein kinase 12 (MPK12; LmxM.29.0370)

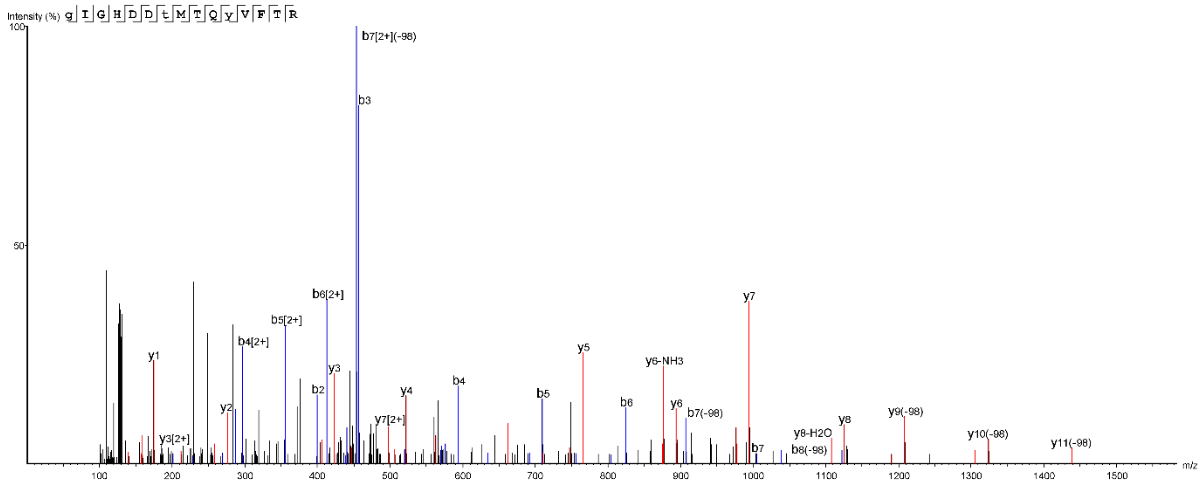


## Mitogen-activated protein kinase 14 (MPK14; LmxM.27.0100)

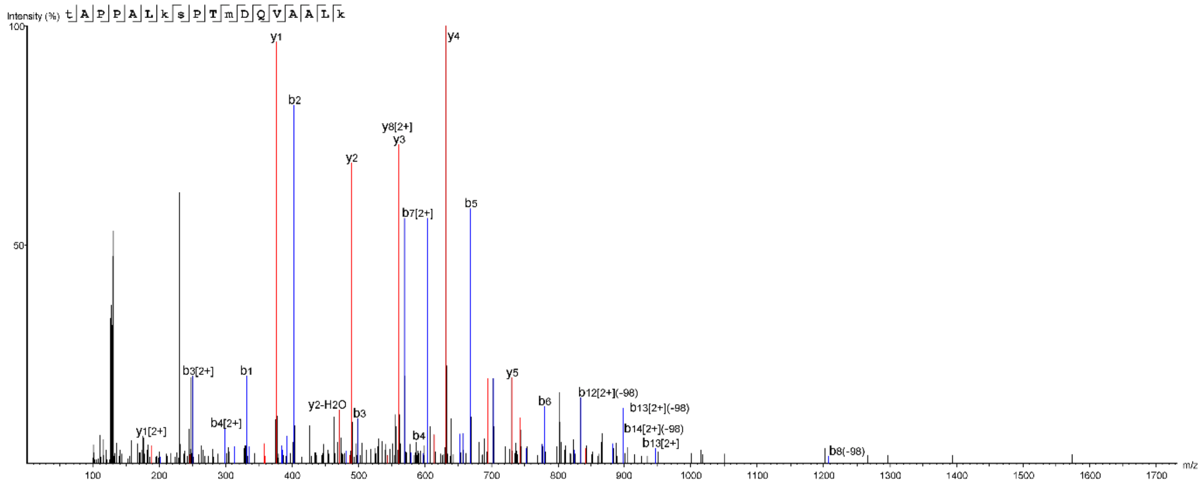

## Mitogen-activated protein kinase 15 (MPK15; LmxM.32.2070)

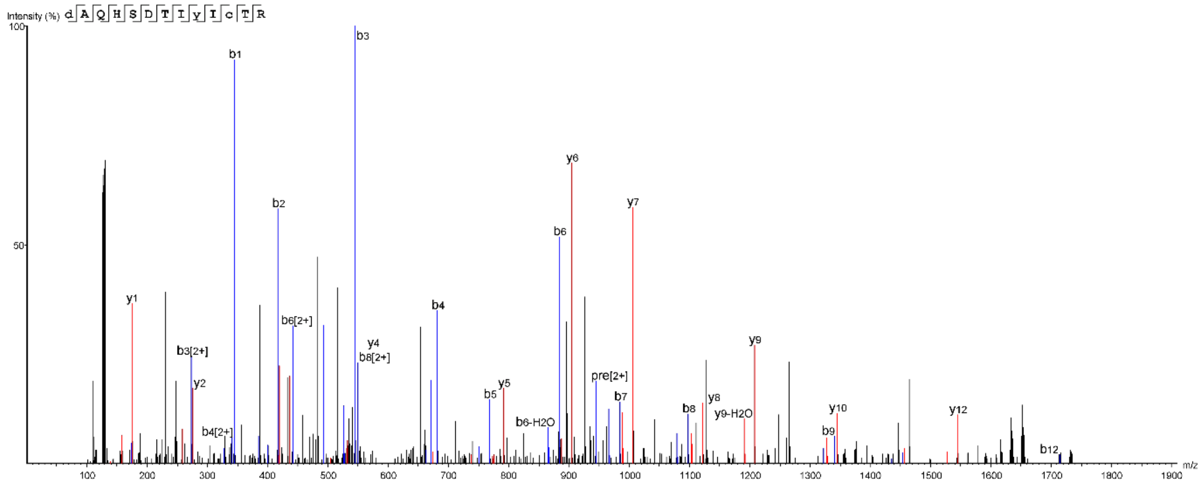


## AGC essential kinase 1 (AEK1; LmxM.25.2340)

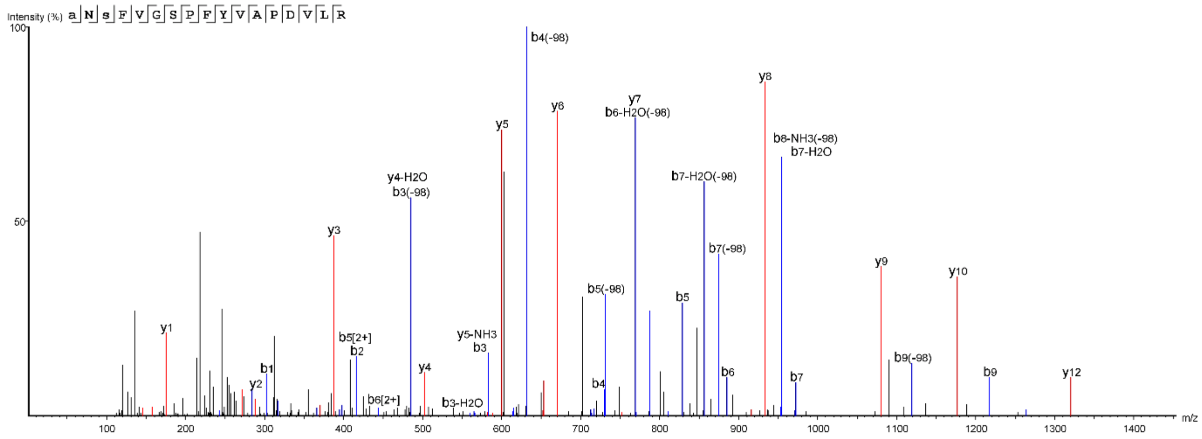


## Mitogen-activated protein kinase kinase 5 (MKK5; LmxM.36.0860)

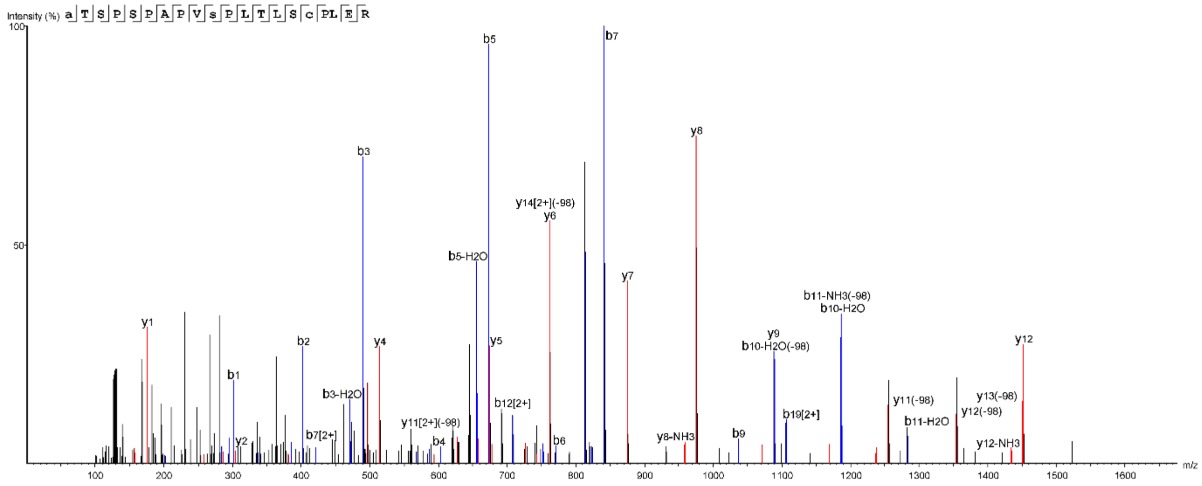

Mitogen-activated protein kinase kinase 7 (MKK7; LmxM.07.0250)

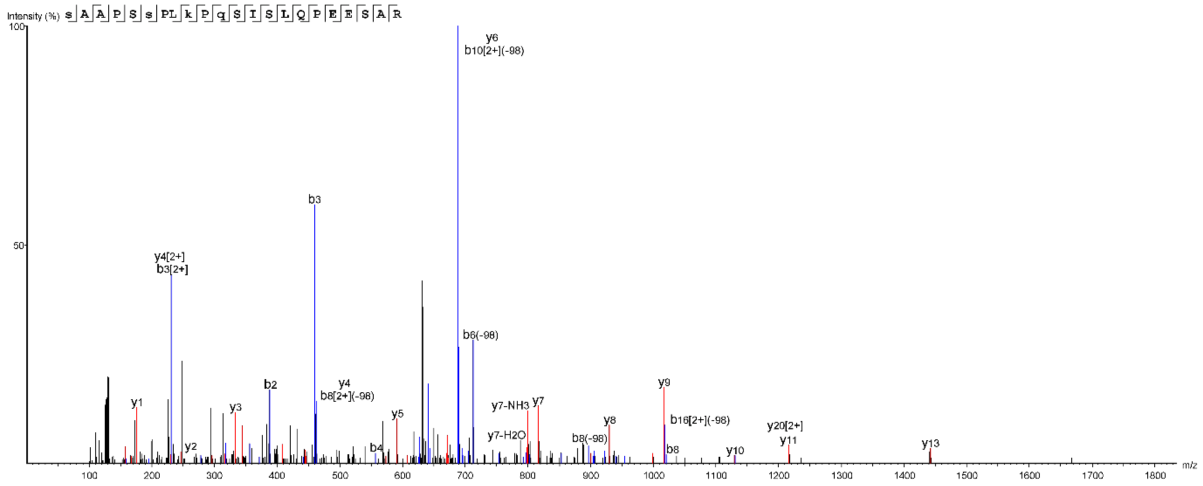


Glycogen synthase kinase 3 beta (GSK3β; LmxM.18.0270)

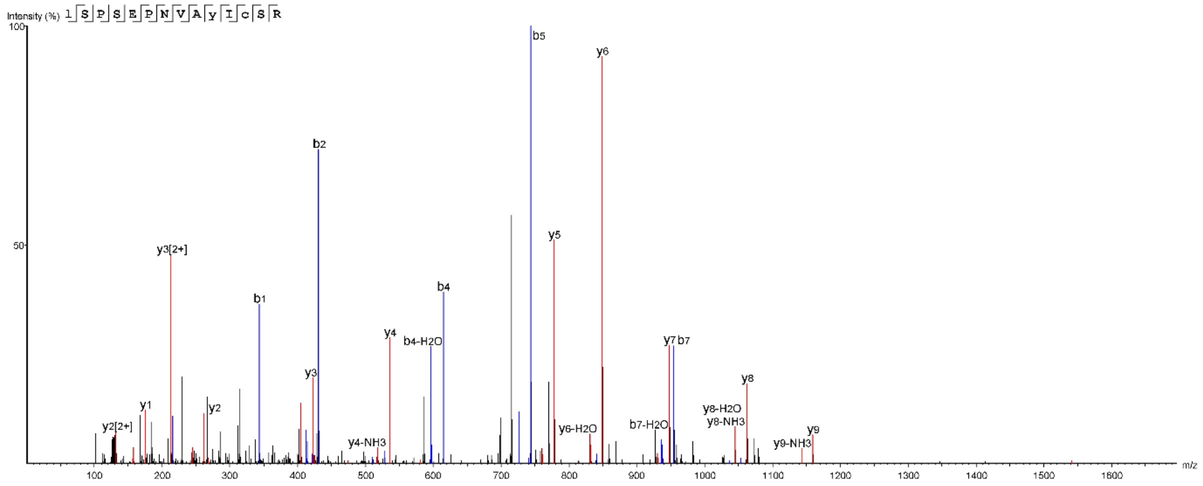


Glycogen synthase kinase 3 beta (GSK3β; LmxM.18.0270)

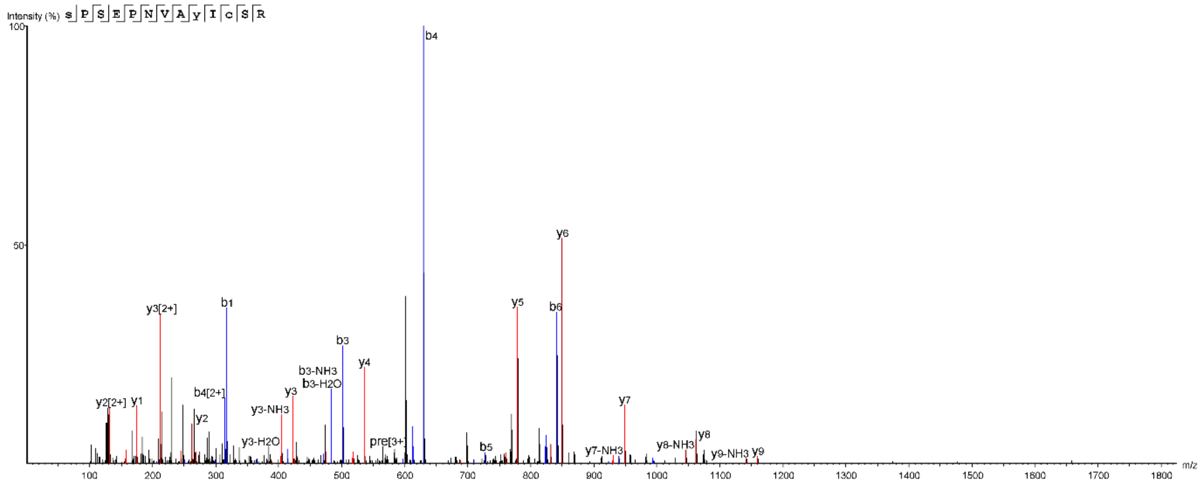

Glycogen synthase kinase 3 beta (GSK3β; LmxM.18.0270)

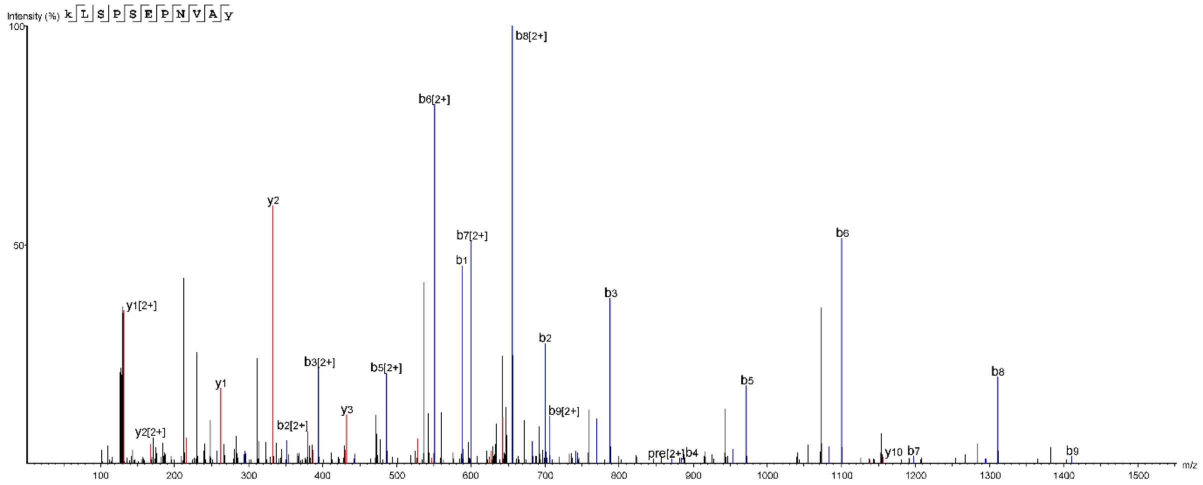


Protein kinase A (PKA; LmxM.34.4010)

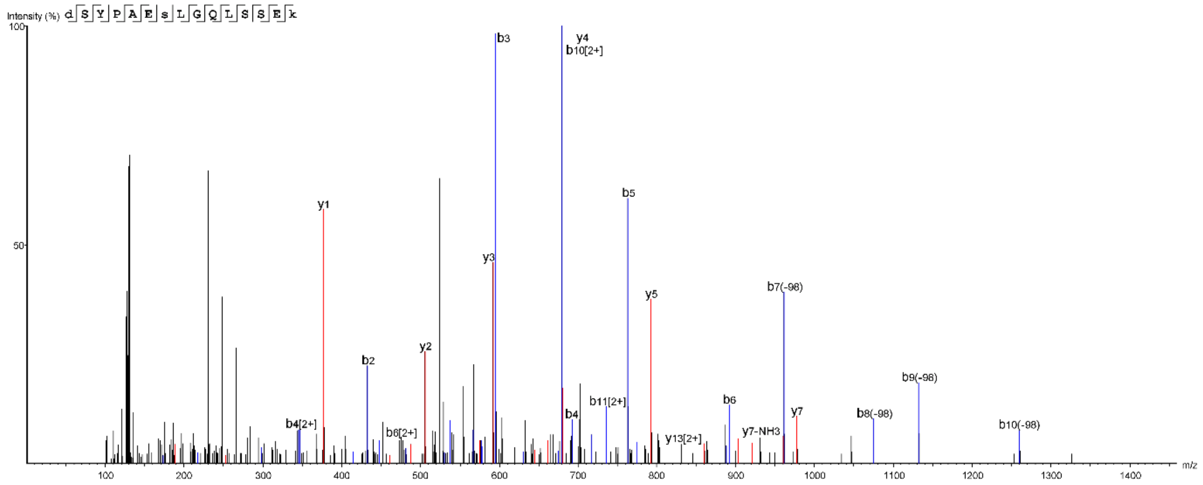


DEATH kinesin (LmxM.29.0350)

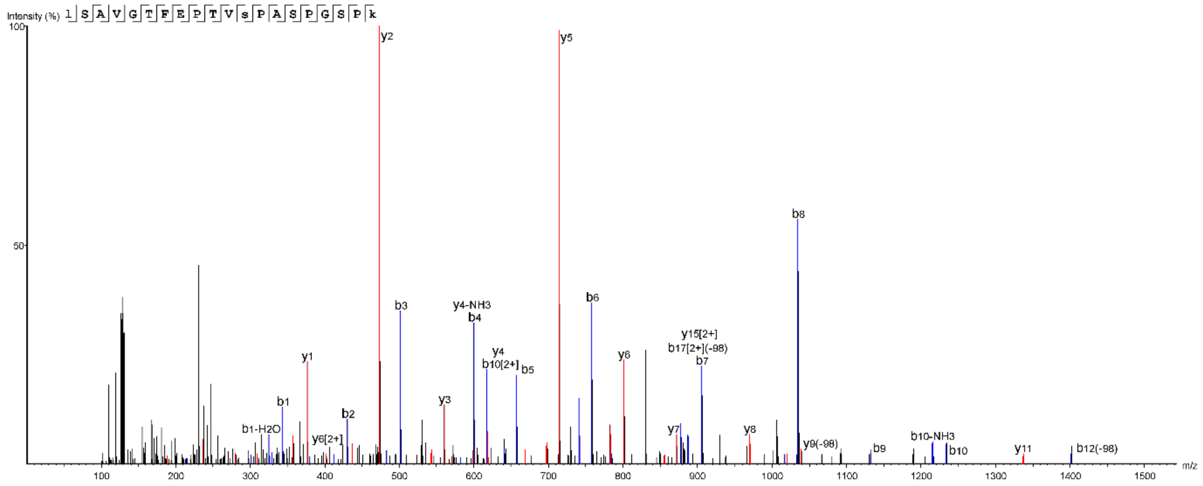

Kinesin D (LmxM.36.5150)

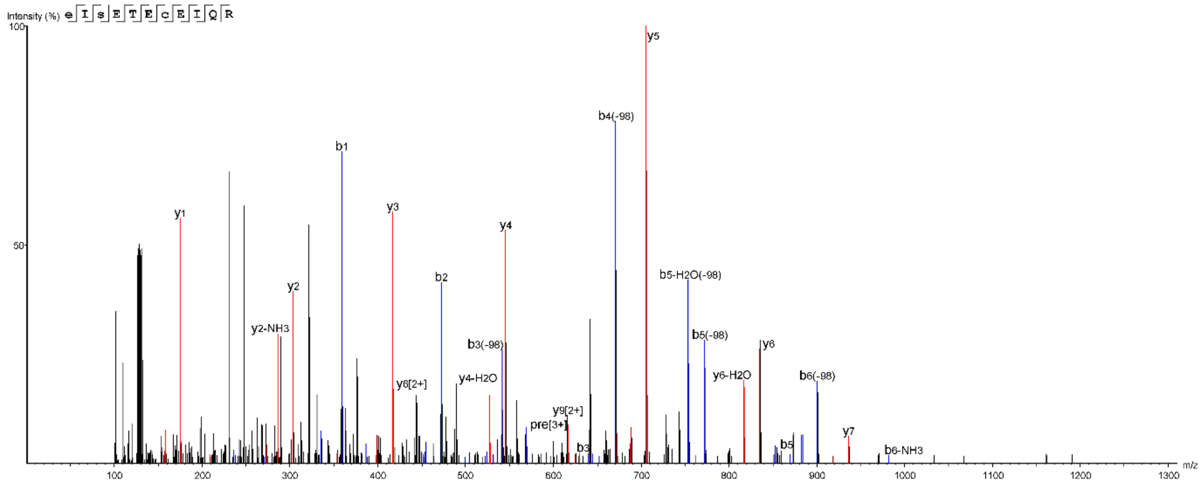


Kinesin (LmxM.11.0870)

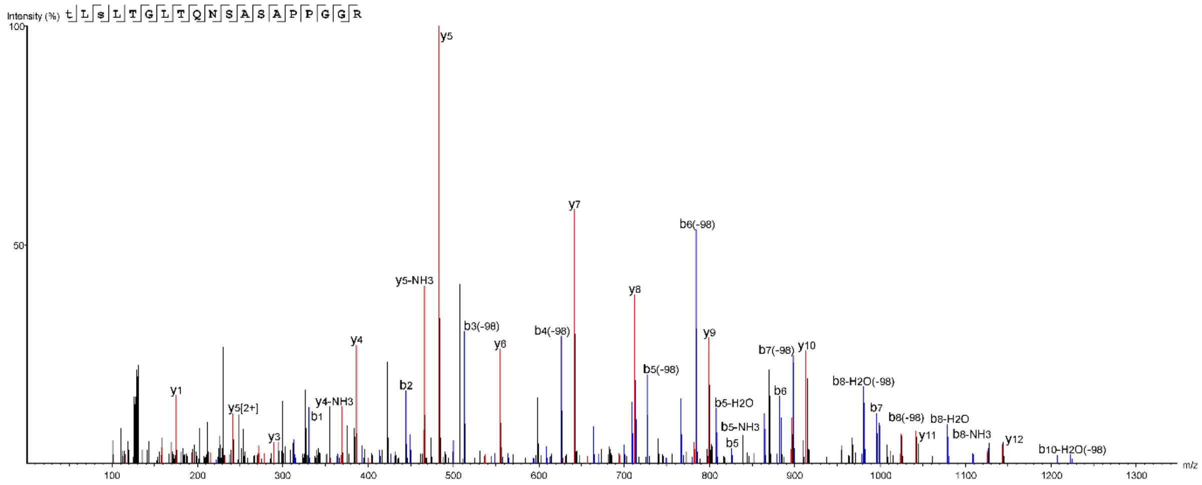


ADP/ATP mitochondrial carrier-like protein (LmxM.14.0990)

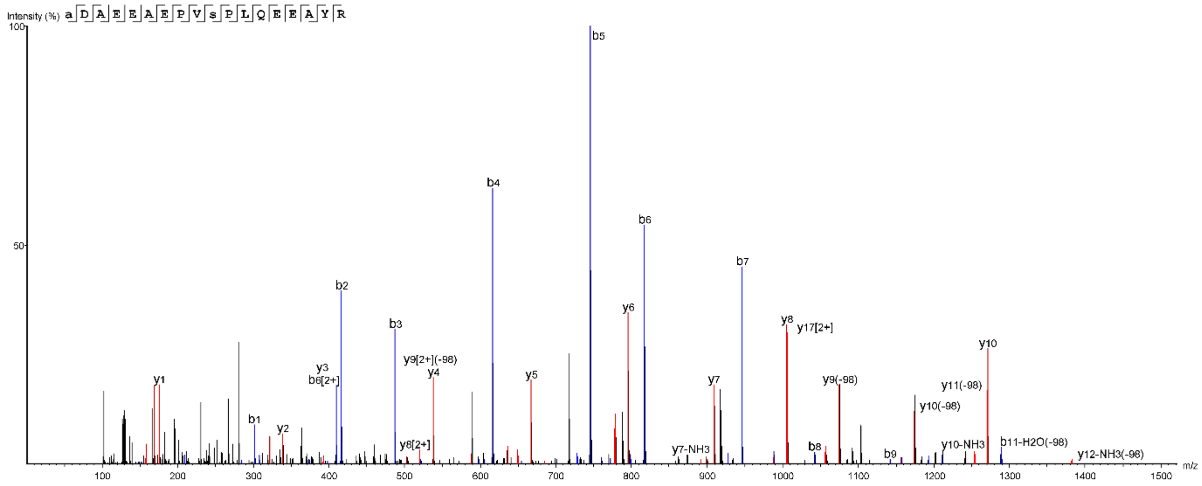

## Isoprenylcysteine alpha-carbonyl methyltransferase (LmxM.15.0320)

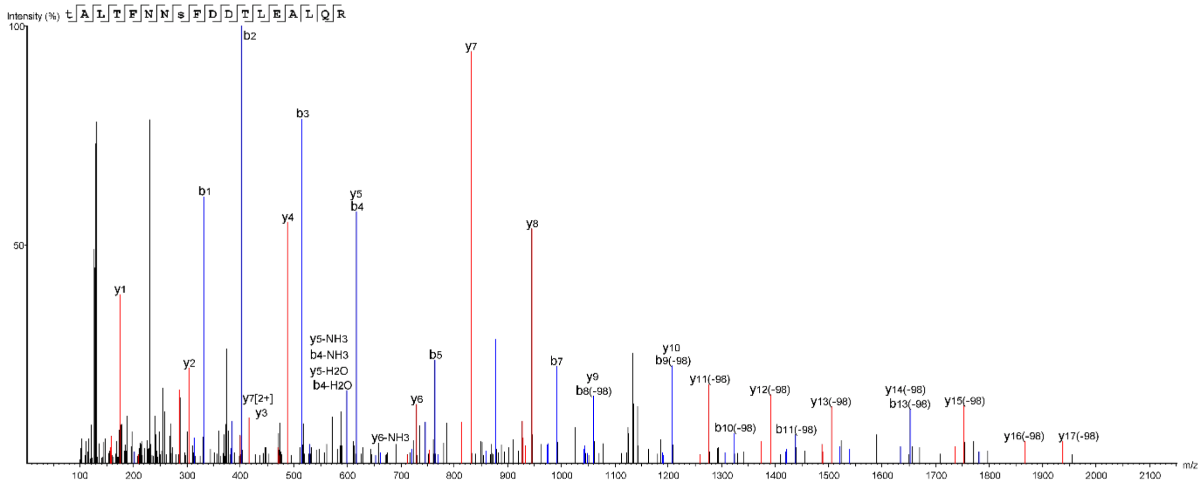


## Carnosine N-methyltransferase (LmxM.33.1020)

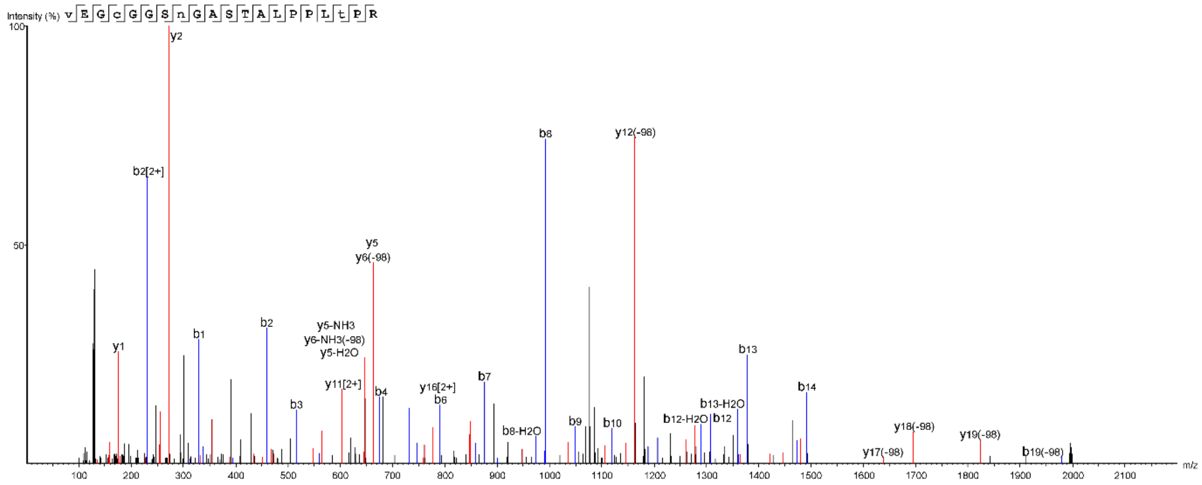


## Distal docking complex protein 1 (LmxM.15.0540)

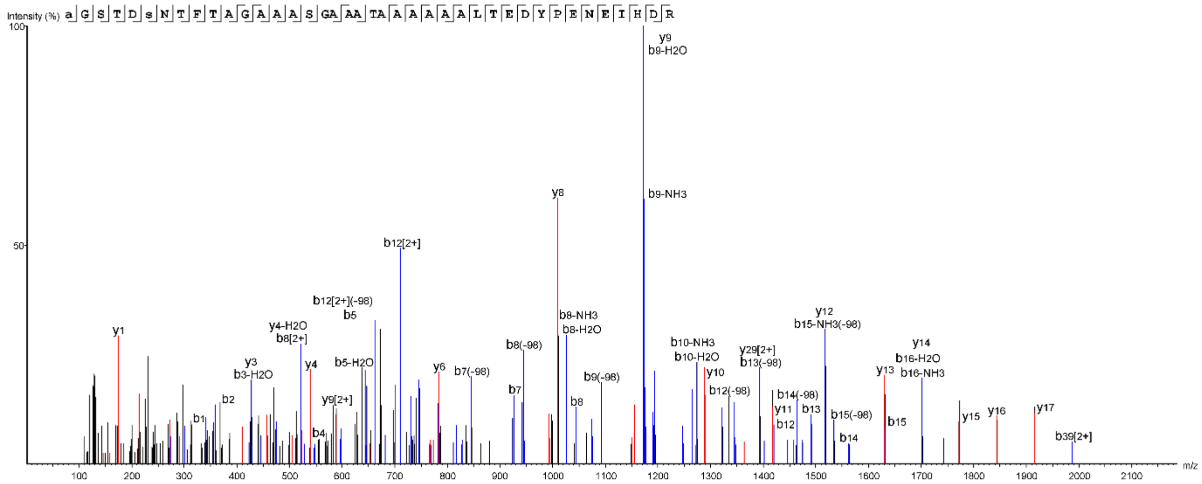

## Cid1 family poly A polymerase (LmxM.19.1410)

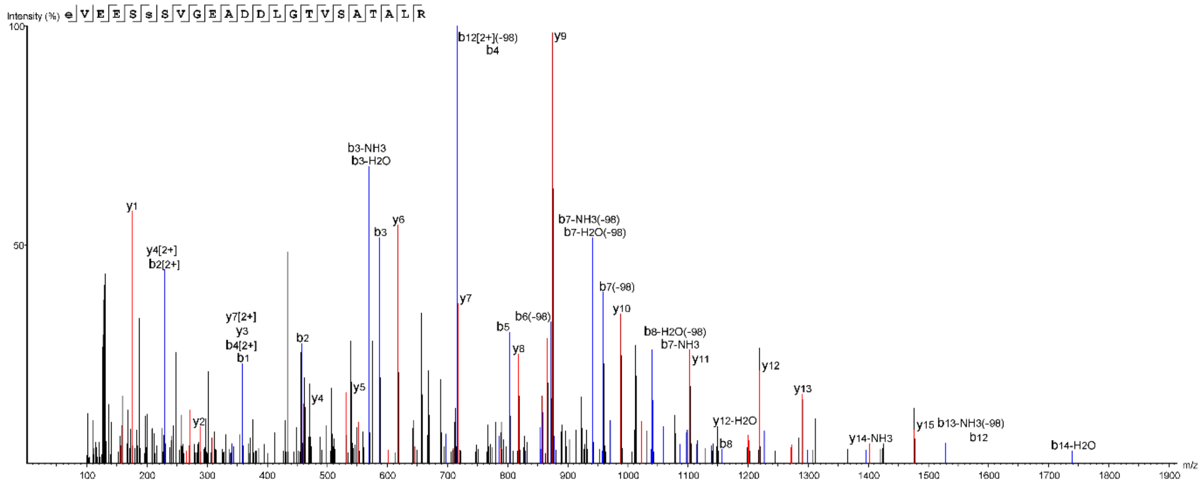


## U3 small nucleolar ribonucleoprotein protein 10 (MPP10; LmxM.08_29.0750)

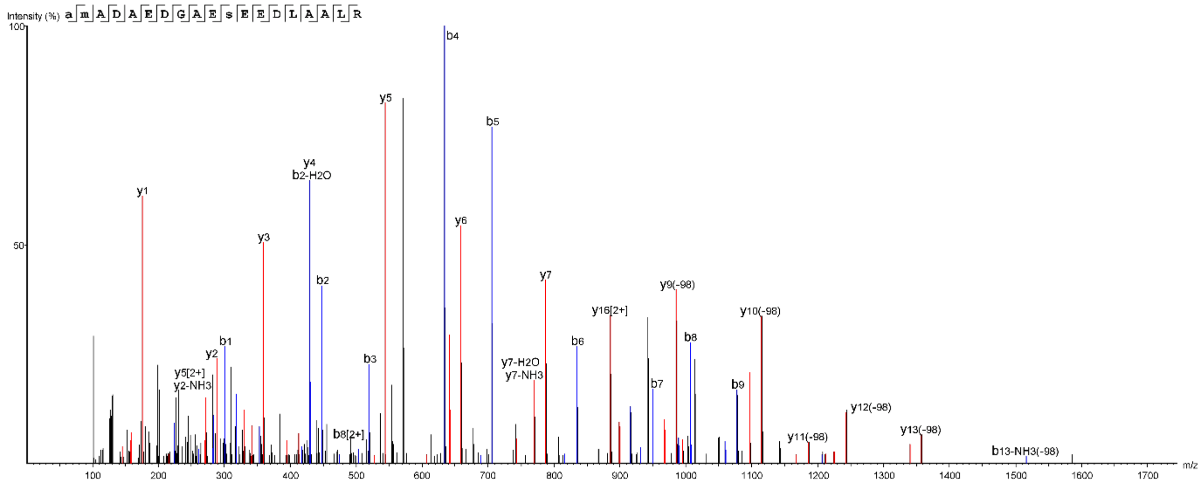


## DEAD/DEAH box helicase (LmxM.03.0690)

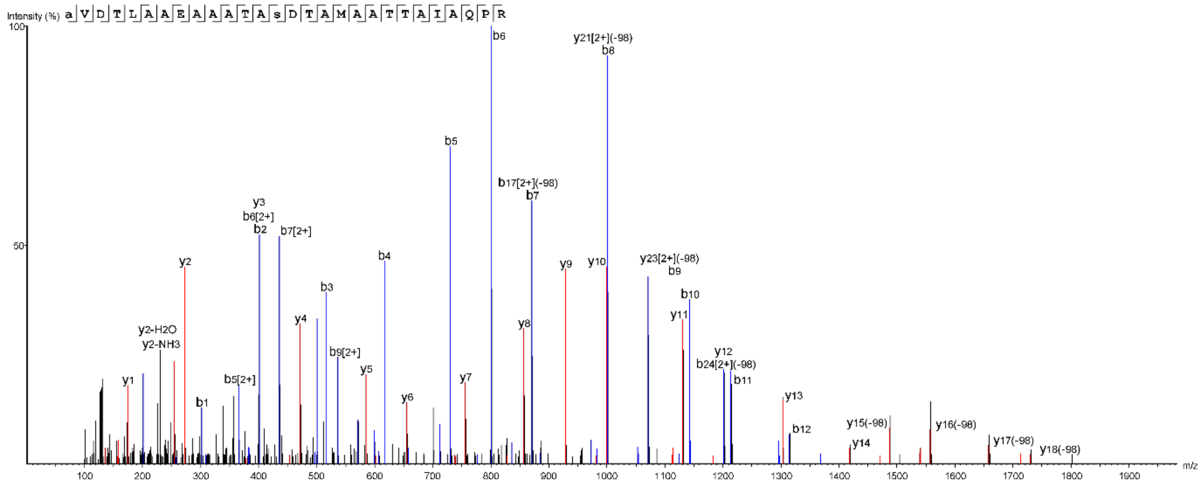

ATP-dependent RNA helicase (LmxM.28.1310)

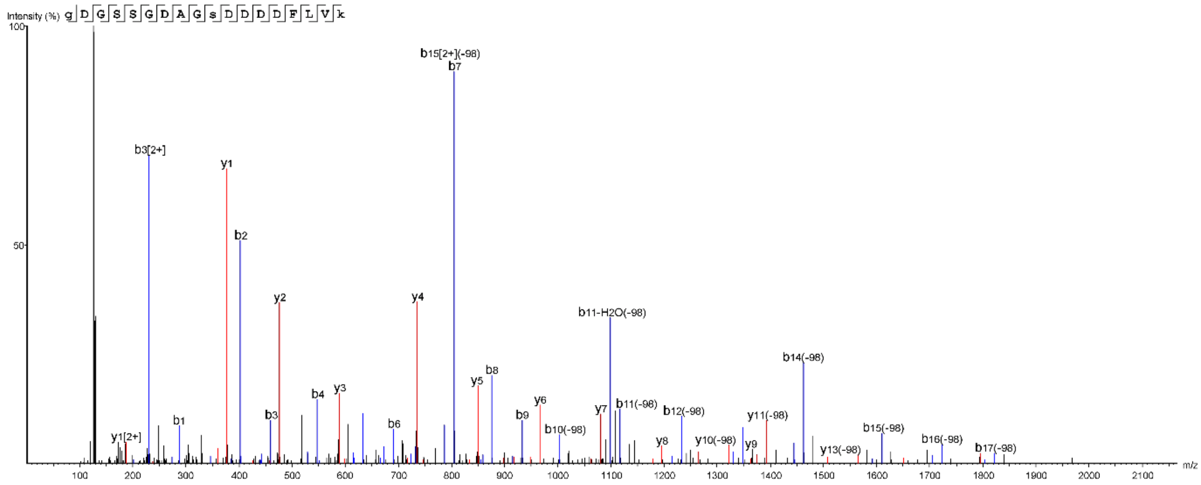


KHARON (LmxM.36.5850)

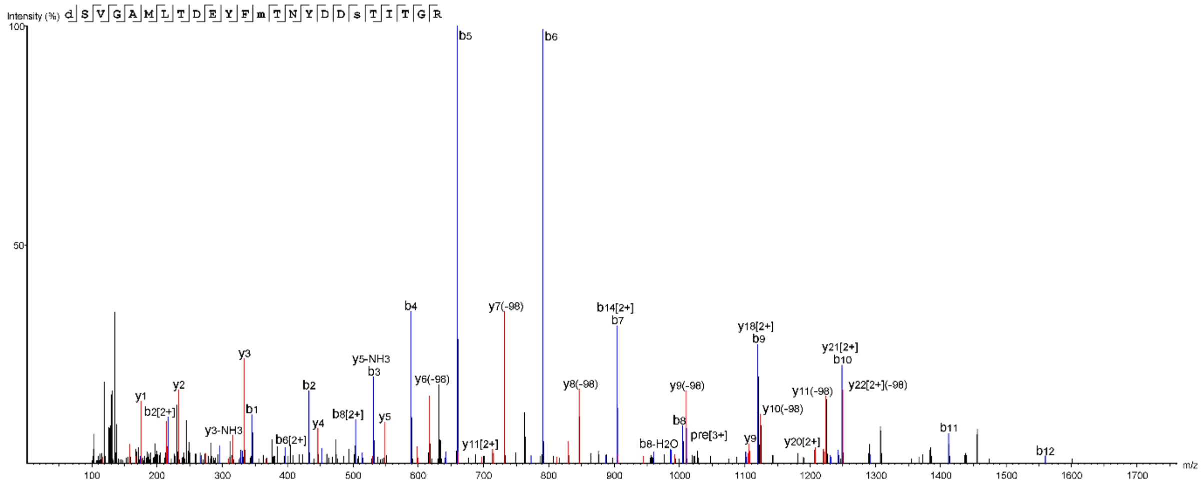


Ubiquitin hydrolase (DUB3; LmxM.29.1200)

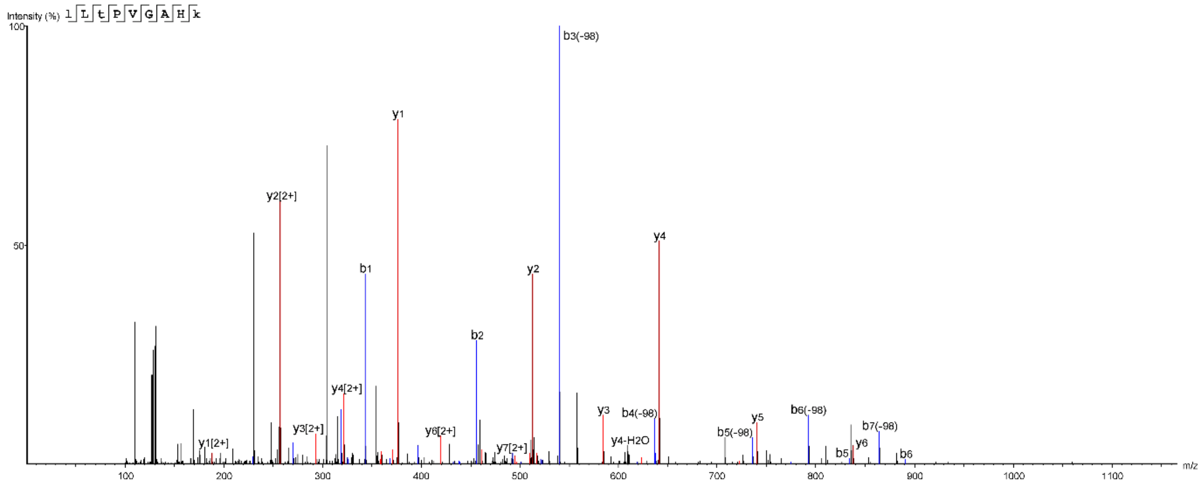

Ubiquitin ligase (LmxM.09.0740)

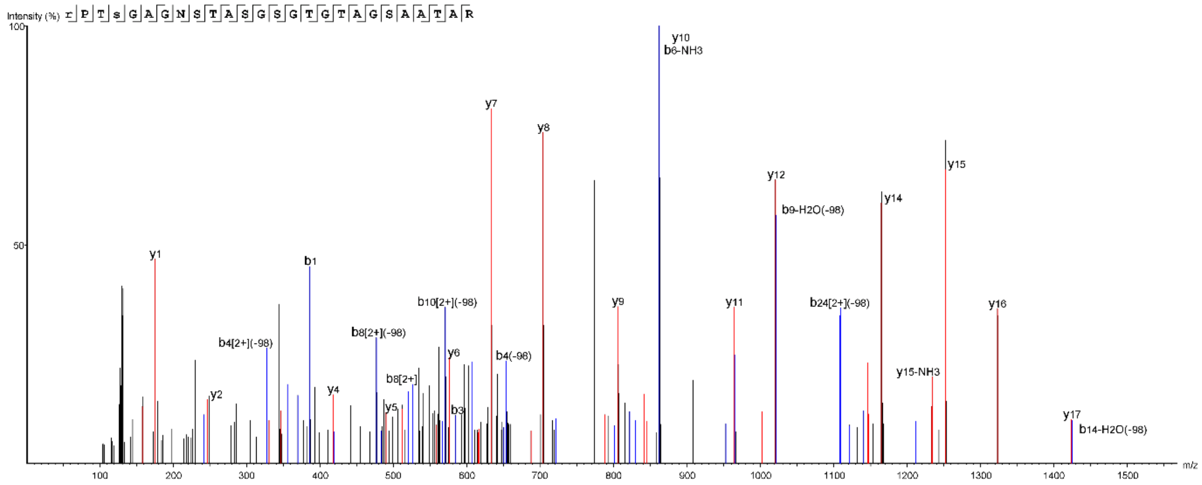


HECT E3 ligase (HECT3; LmxM.07.0280)

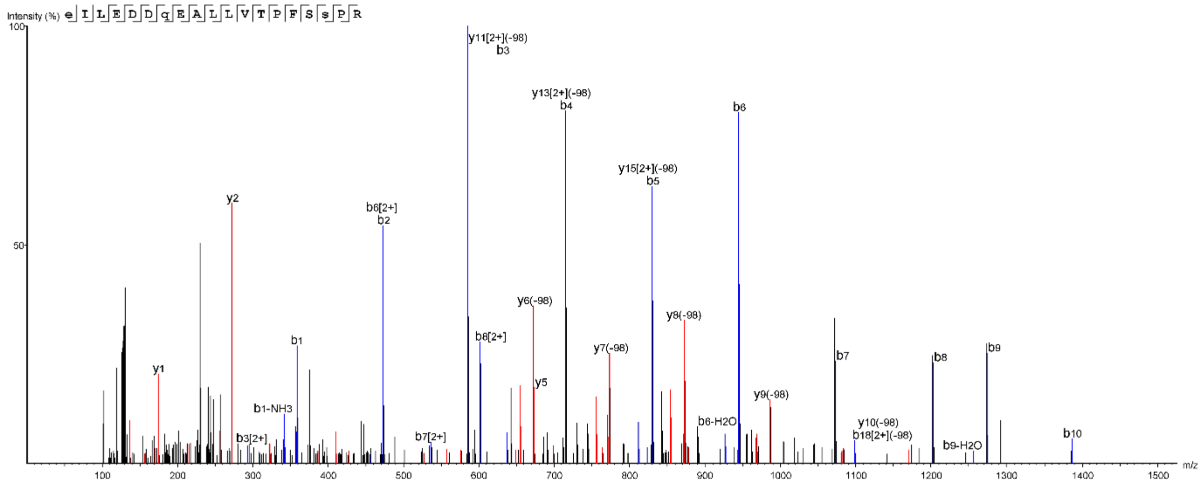


HECT E3 ligase (HECT7; LmxM.31.1090)

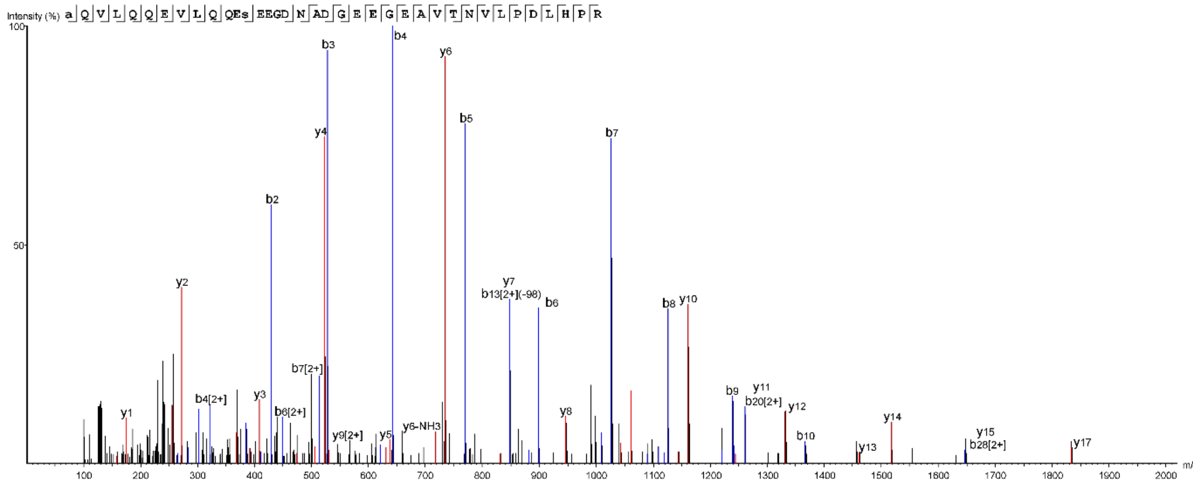


**FIG. S2** MS/MS spectra of phosphorylated tryptic peptides from selected *L. mexicana* proteins.

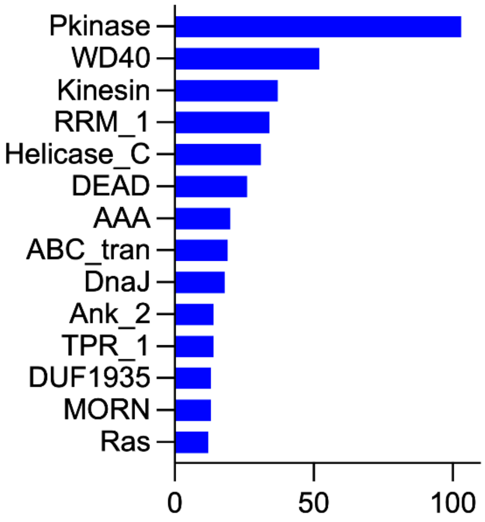


**FIG. S3** Protein families and domains (Pfam) annotation bar chart of *L. mexicana* phosphoproteome. Top 14 Pfam annotations based on the number of phosphoproteins identified are shown.

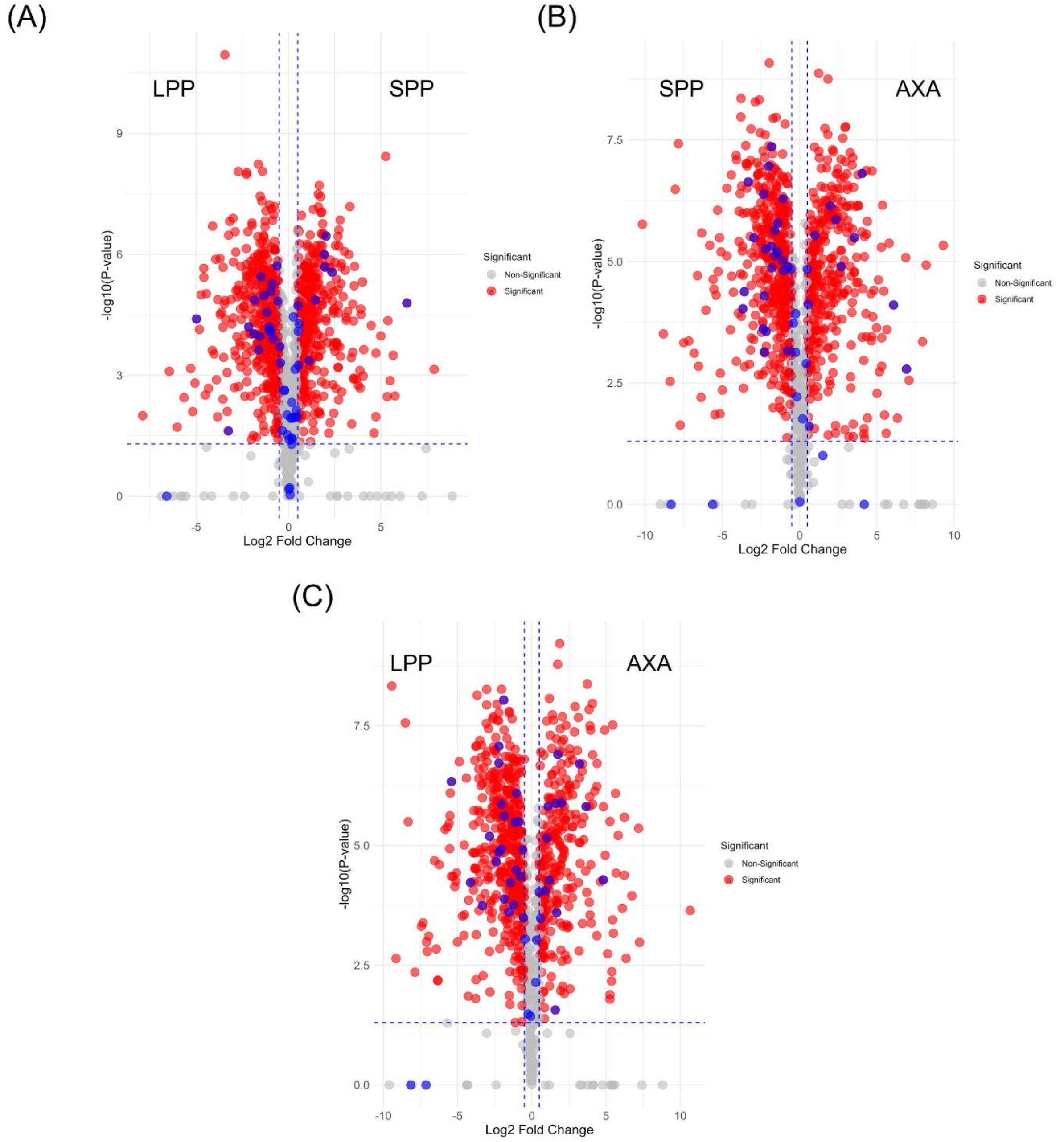


**FIG. S4** Differential expression of phosphoproteins across log phase promastigote (LPP), stationary phase promastigote (SPP) and axenic amastigote (AXA) life cycle stages of *L. mexicana*. LPP vs SPP (A) SPP vs AXA (B) and LPP vs AXA (C) volcano plots with protein kinases highlighted in blue.

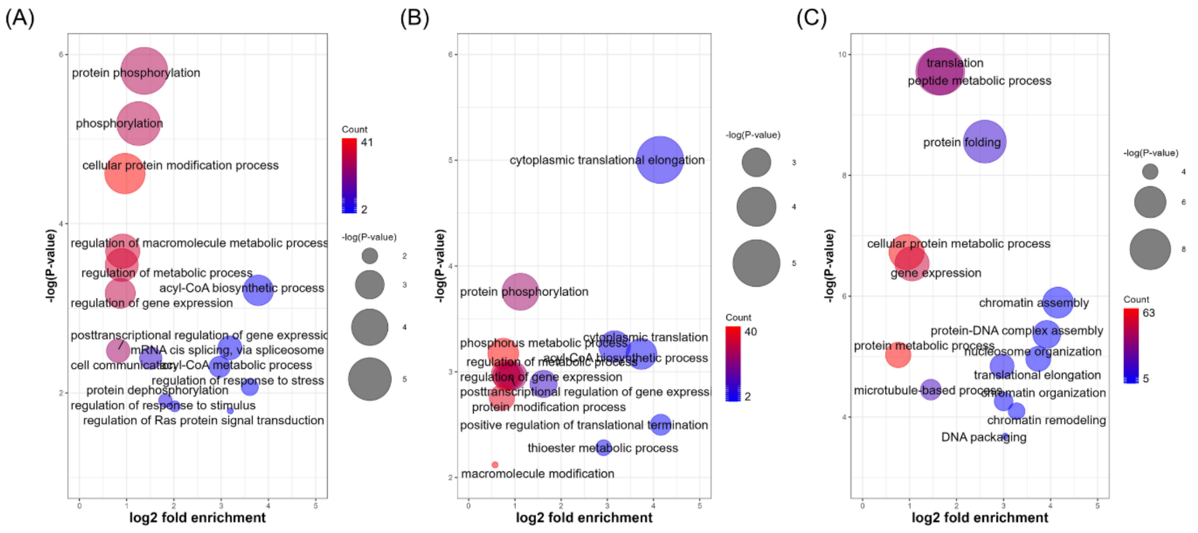


**FIG. S5** Biological Process (BP) gene ontology (GO) terms enriched in (A) log phase promastigote (LPP), (B) stationary phase promastigote (SPP) and (C) axenic amastigote (AXA) phosphoproteins.

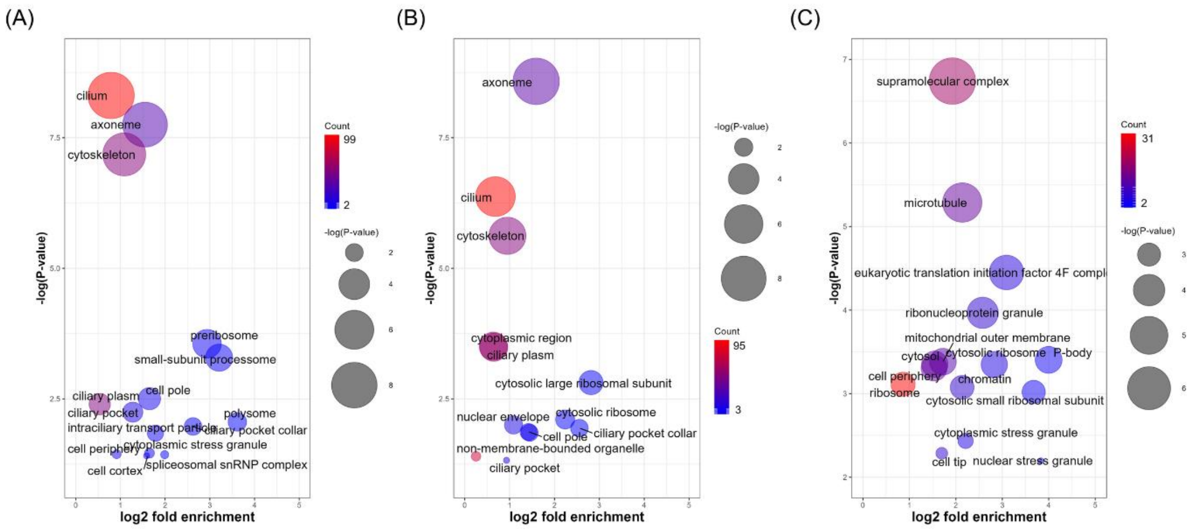


**FIG. S6** Cellular Component (CC) gene ontology (GO) terms enriched in (A) log phase promastigote (LPP), (B) stationary phase promastigote (SPP) and (C) axenic amastigote (AXA) phosphoproteins.

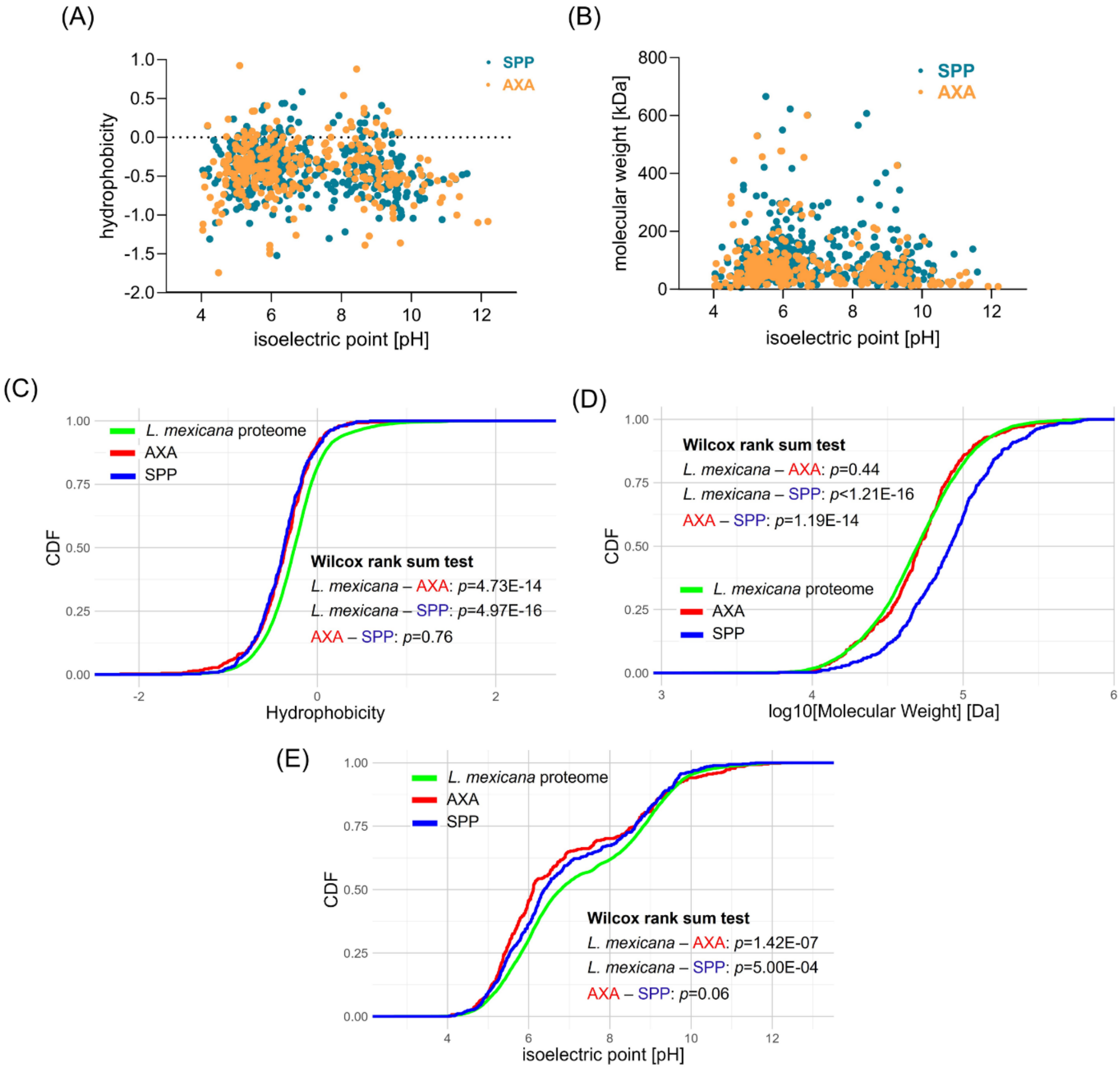


**FIG. S7** Physicochemical properties of *L. mexicana* phosphorylation substrates. (A) and (B) Scatter plots comparing hydrophobicity and isoelectric points and molecular weights and isoelectric points respectively of phosphorylation substrates in stationary phase promastigote (SPP) and axenic amastigote (AXA) life cycle stages. (C), (D) and (E) Cumulative distributions of hydrophobicity, molecular weights and isoelectric points respectively in the AXA and SPP phosphorylation substrates and the entire *L. mexicana* proteome. Wilcox rank sum test *p* values of the comparisons *L. mexicana* total proteome vs AXA phosphorylation substrates (*L. mexicana* – AXA) , *L. mexicana* total proteome vs SPP phosphorylation substrates (*L. mexicana* – SPP) and AXA vs SPP phosphorylation substrates (AXA – SPP) are shown.

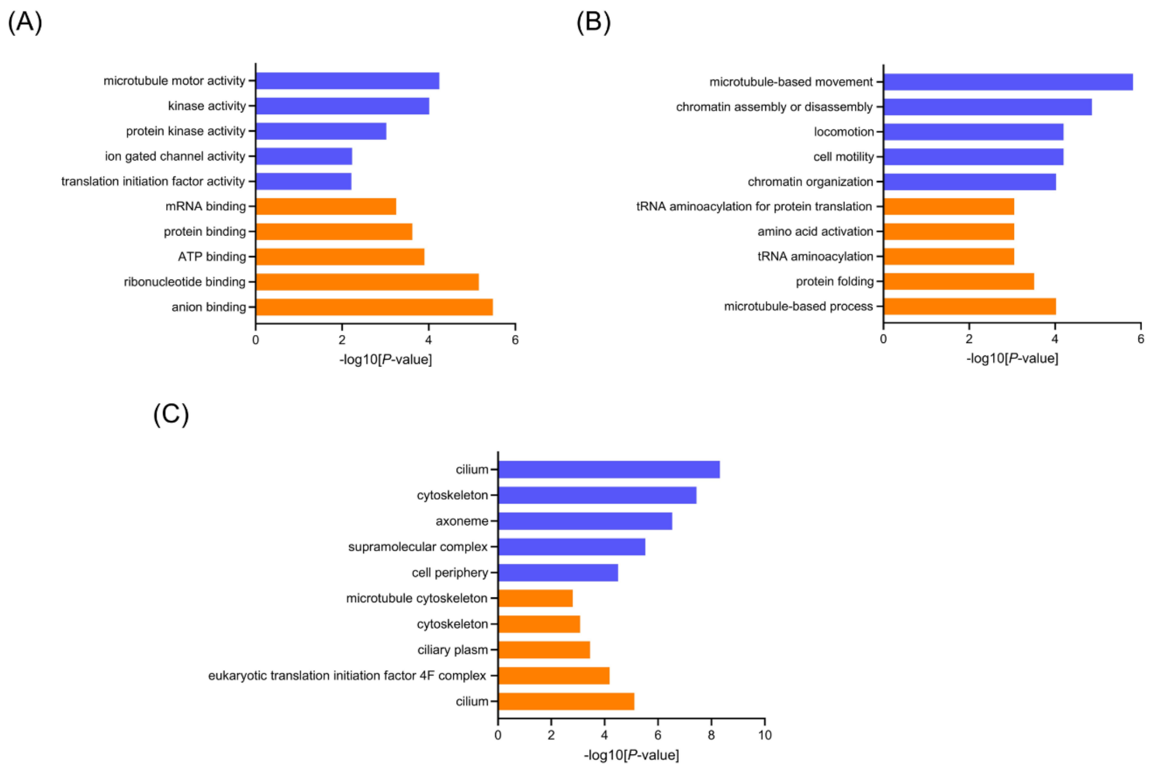


**FIG. S8** GO analysis of tanespimycin-affected phosphoproteins in *L. mexicana* log phase promastigotes (LPPs). (A), (B) and (C) are Molecular Function (MF), Biological Process (BP) and Cellular Component (CC) GO terms respectively; blue: negatively affected phosphoproteins, red: positively affected phosphoproteins.

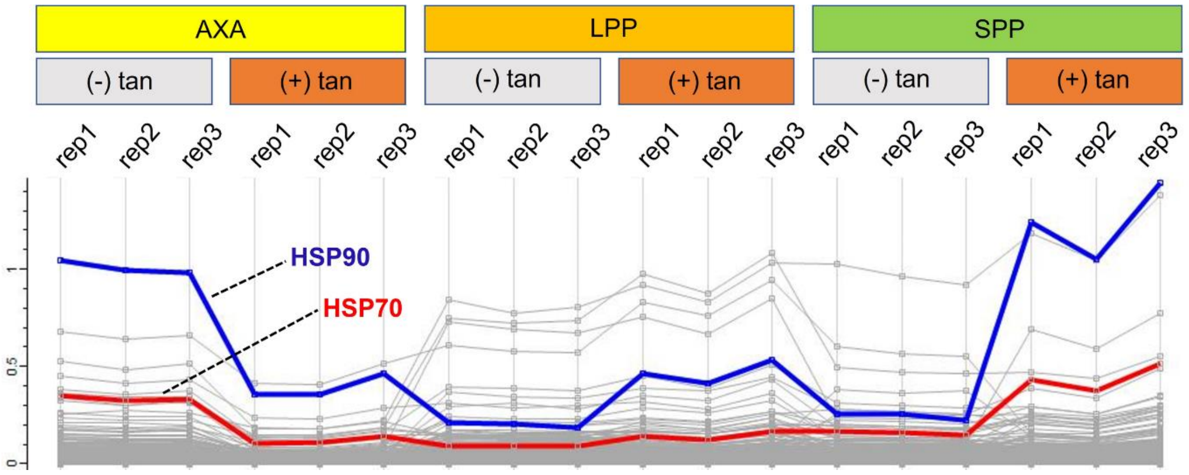


**FIG. S9** Profile plot of the *L. mexicana* phosphoproteins with (+) and without (-) tanespimycin (tan) treatment in axenic amastigote (AXA), log phase promastigote (LPP) and stationary phase promastigote (SPP) life cycle stages. HSP90 (LmxM.32.0316) and HSP70 (LmxM.28.2770) proteins are highlighted in blue and red respectively.

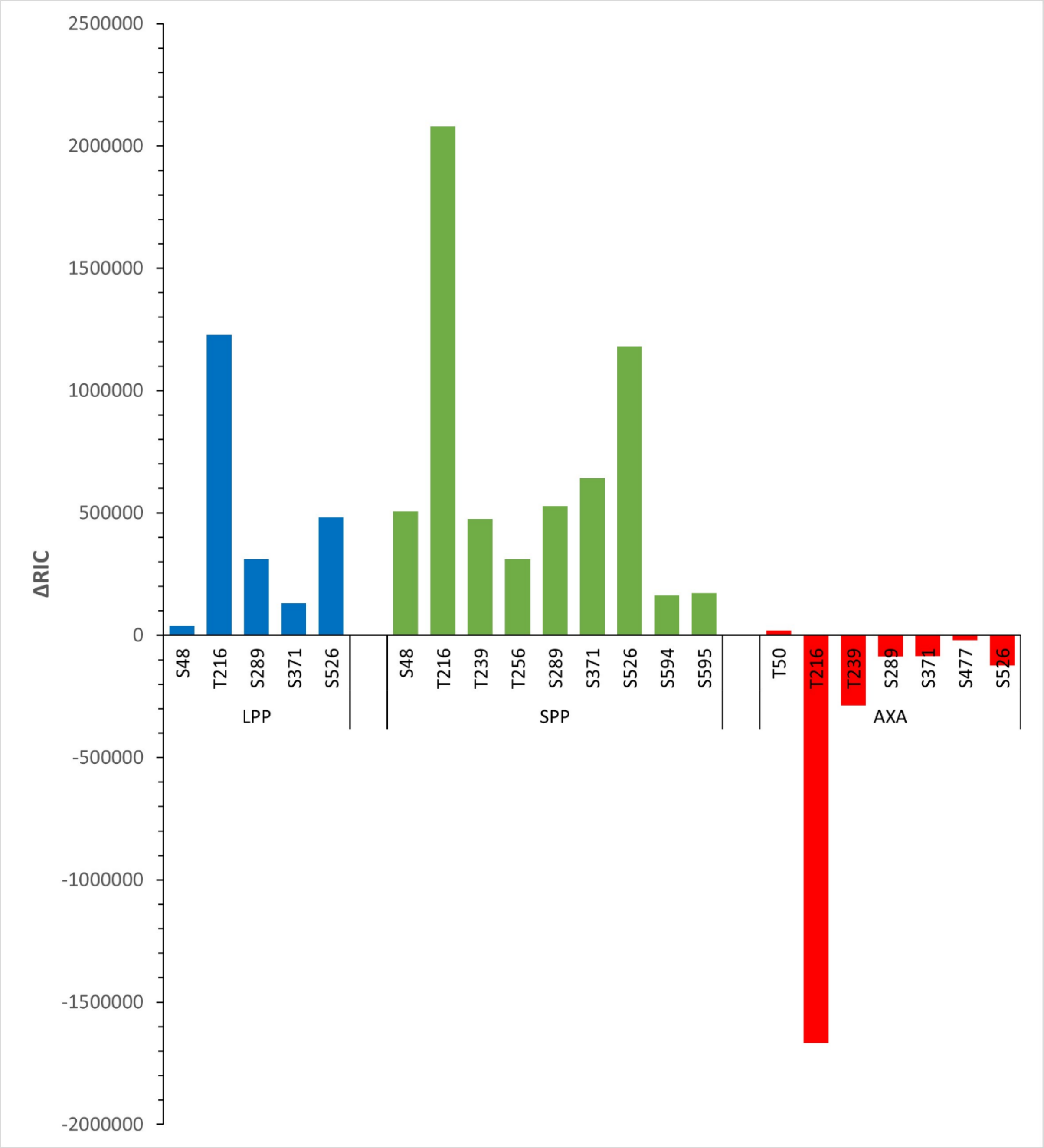


**FIG. S10** Changes in phosphorylation levels detected in *L. mexicana* HSP90 phosphorylation sites across its log phase promastigote (LPP), stationary phase promastigote (SPP) and axenic amastigote (AXA) life cycle stages upon treatment with tanespimycin. Mean difference of reporter intensity corrected values (ΔRIC) between tanespimycin treatment and no treatment for all detected phosphopeptides at each modification site is shown.

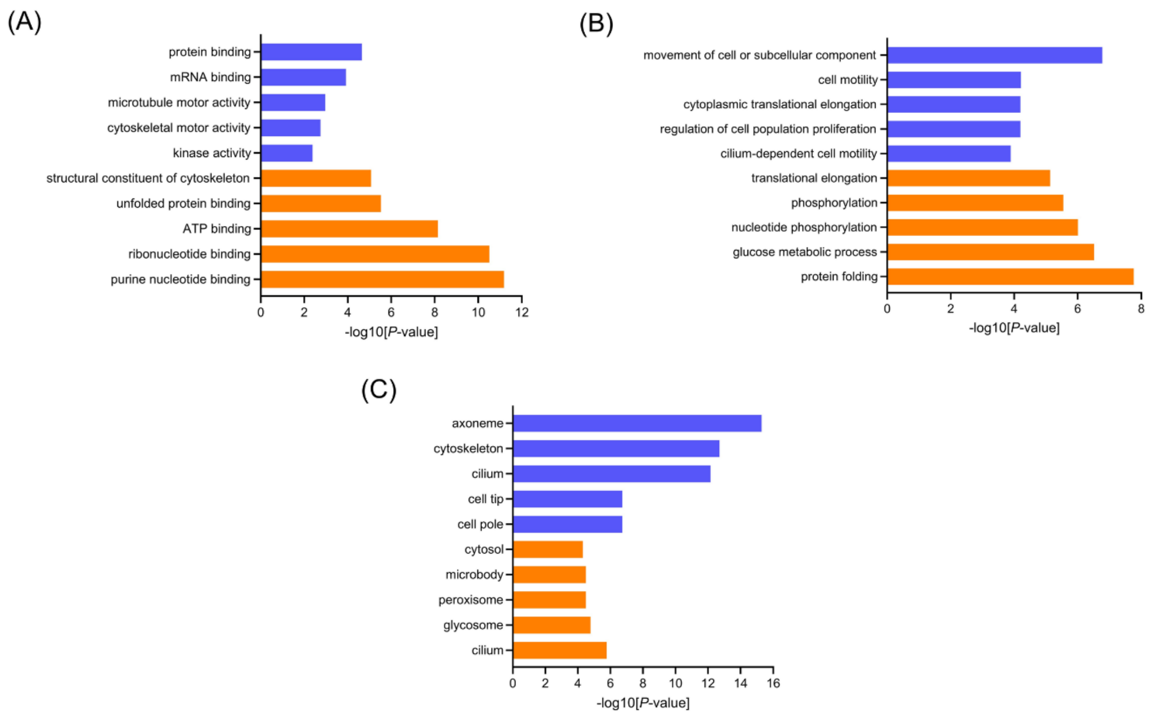


**FIG. S11** GO analysis of tanespimycin-affected phosphoproteins in *L. mexicana* stationary phase promastigotes (SPPs). (A), (B) and (C) are Molecular Function (MF), Biological Process (BP) and Cellular Component (CC) GO terms respectively; blue: negatively affected phosphoproteins, red: positively affected phosphoproteins.

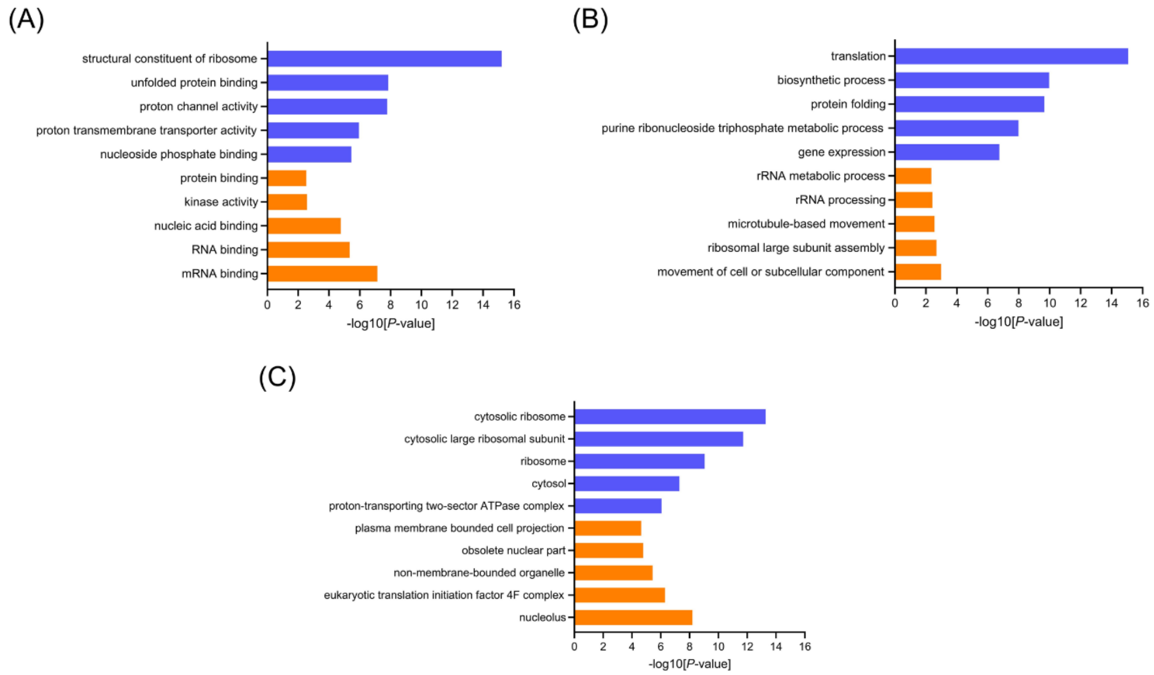

**FIG. S12** GO analysis of tanespimycin-affected phosphoproteins in *L. mexicana* axenic amastigotes (AXAs). (A), (B) and (C) are Molecular Function (MF), Biological Process (BP) and Cellular Component (CC) GO terms respectively; blue: negatively affected phosphoproteins, red: positively affected phosphoproteins.

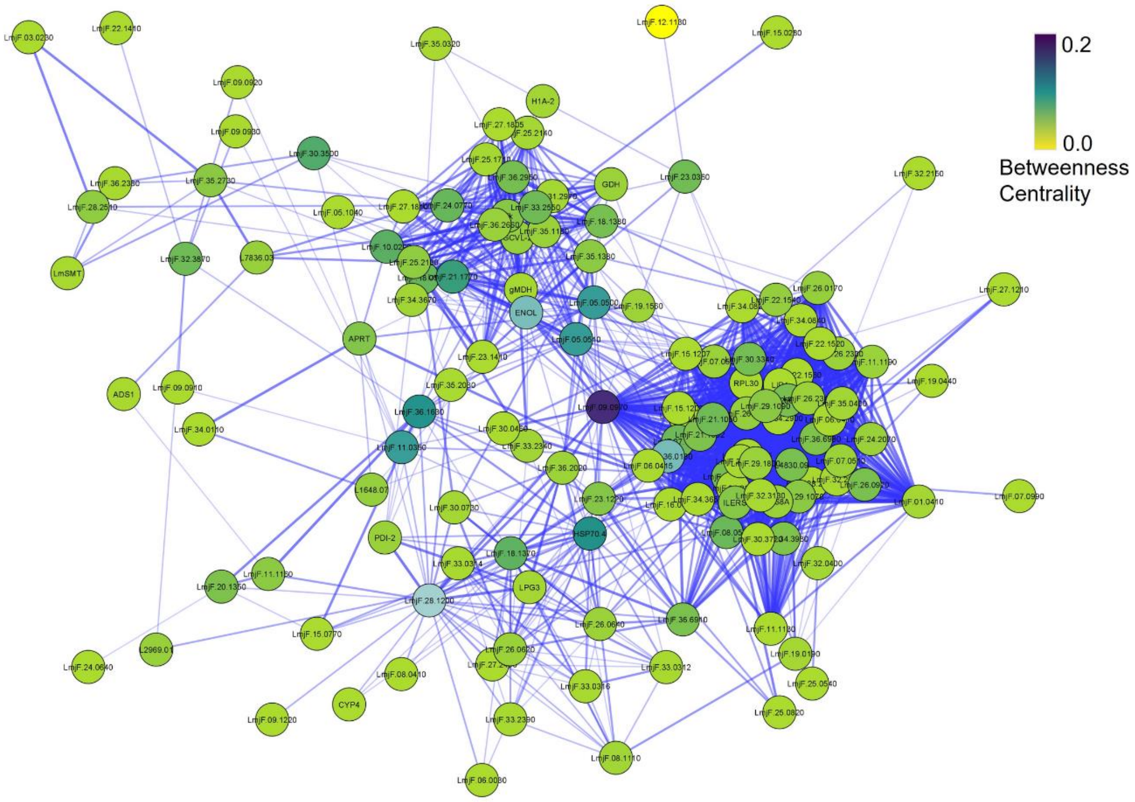


**FIG. S13** Protein-protein interaction network of RNA-binding phosphoproteins downregulated during HSP90 inhibition in *L. mexicana* axenic amastigotes (AXAs) constructed using publicly available STRING database of *L. major* Friedlin strain. The nodes are coloured according to their betweenness centrality in the network.

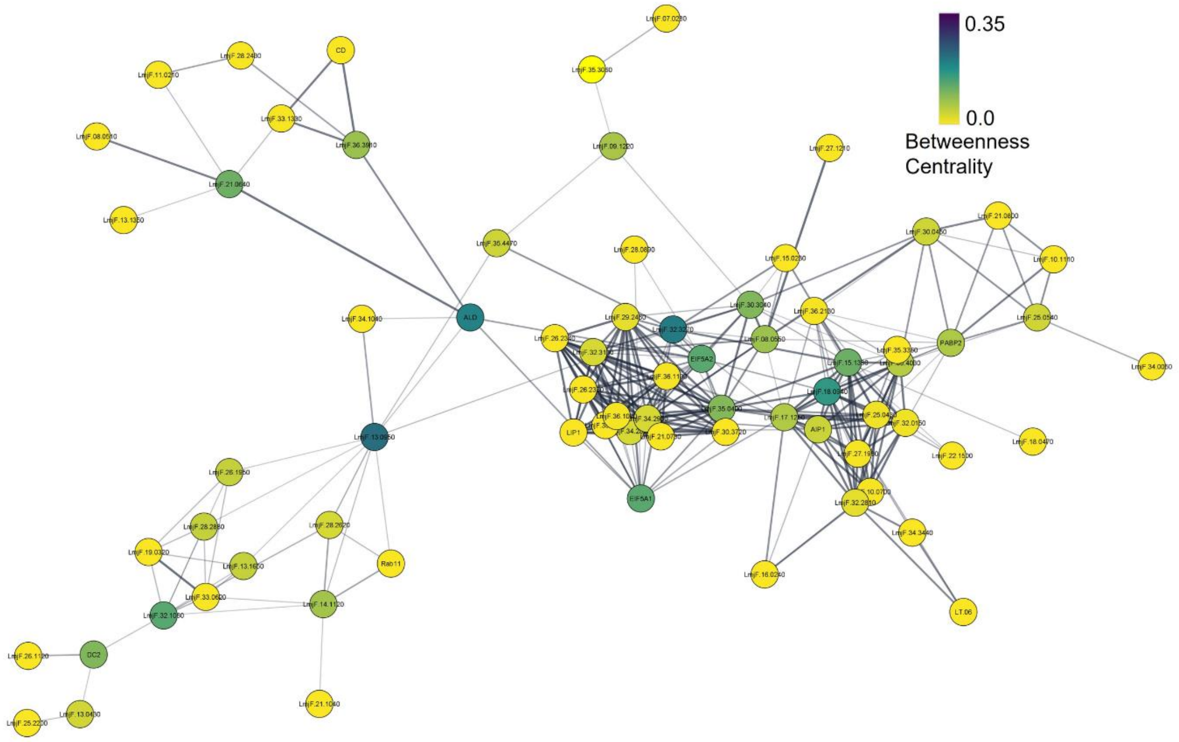


**FIG. S14** Protein-protein interaction network of RNA-binding phosphoproteins downregulated during HSP90 inhibition in *L. mexicana* log phase promastigotes (LPPs) constructed using publicly available STRING database of *L. major* Friedlin strain. The nodes are coloured according to their betweenness centrality in the network.

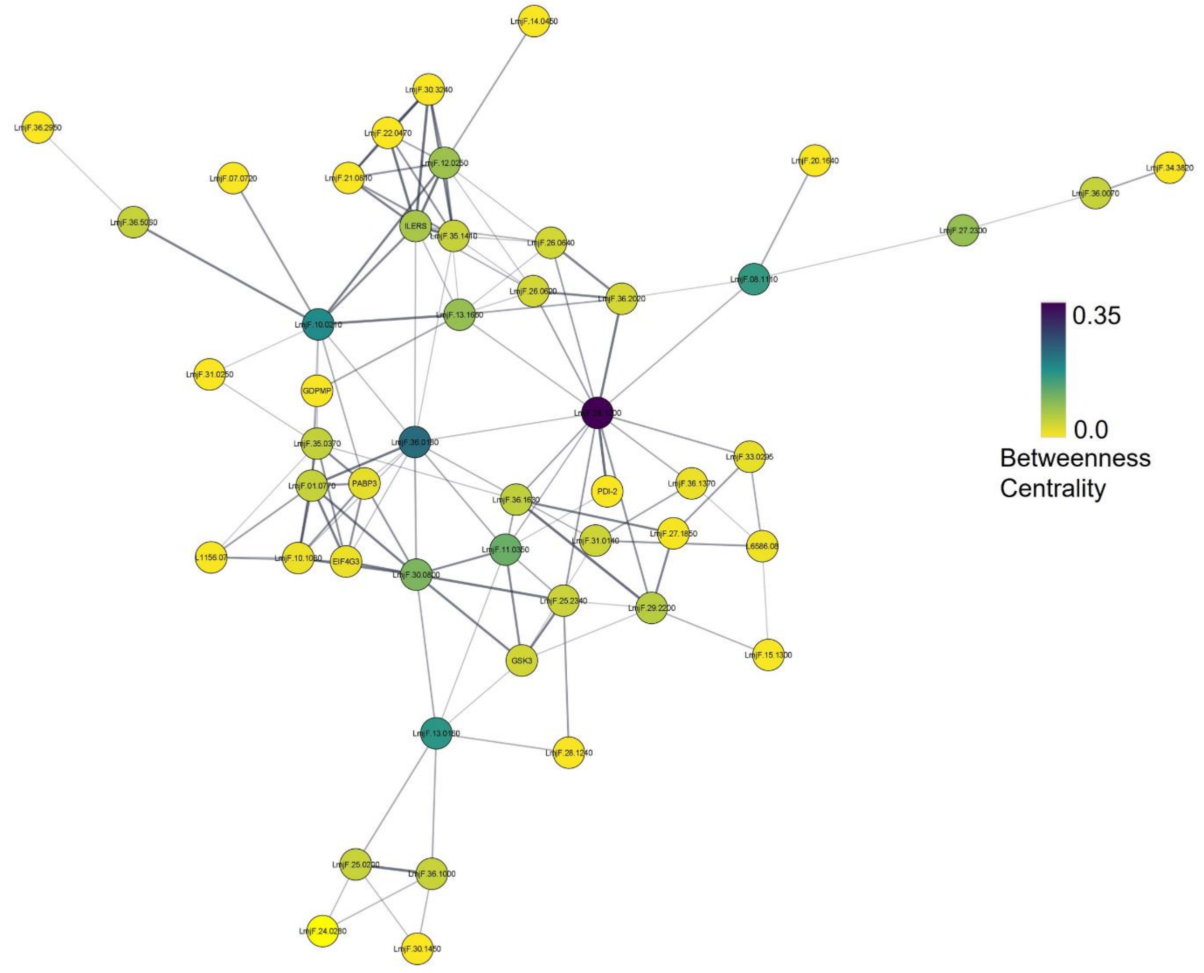


**FIG. S15** Protein-protein interaction network of RNA-binding phosphoproteins upregulated during HSP90 inhibition in *L. mexicana* log phase promastigotes (LPPs) constructed using publicly available STRING database of *L. major* Friedlin strain. The nodes are coloured according to their betweenness centrality in the network.
